# 14 examples of how LLMs can transform materials science and chemistry: a reflection on a large language model hackathon†

**DOI:** 10.1039/d3dd00113j

**Published:** 2023-08-08

**Authors:** Kevin Maik Jablonka, Qianxiang Ai, Alexander Al-Feghali, Shruti Badhwar, Joshua D. Bocarsly, Andres M. Bran, Stefan Bringuier, L. Catherine Brinson, Kamal Choudhary, Defne Circi, Sam Cox, Wibe A. de Jong, Matthew L. Evans, Nicolas Gastellu, Jerome Genzling, María Victoria Gil, Ankur K. Gupta, Zhi Hong, Alishba Imran, Sabine Kruschwitz, Anne Labarre, Jakub Lála, Tao Liu, Steven Ma, Sauradeep Majumdar, Garrett W. Merz, Nicolas Moitessier, Elias Moubarak, Beatriz Mouriño, Brenden Pelkie, Michael Pieler, Mayk Caldas Ramos, Bojana Ranković, Samuel G. Rodriques, Jacob N. Sanders, Philippe Schwaller, Marcus Schwarting, Jiale Shi, Berend Smit, Ben E. Smith, Joren Van Herck, Christoph Völker, Logan Ward, Sean Warren, Benjamin Weiser, Sylvester Zhang, Xiaoqi Zhang, Ghezal Ahmad Zia, Aristana Scourtas, K. J. Schmidt, Ian Foster, Andrew D. White, Ben Blaiszik

**Affiliations:** a Laboratory of Molecular Simulation (LSMO), Institut des Sciences et Ingénierie Chimiques, Ecole Polytechnique Fédérale de Lausanne (EPFL) Sion Valais Switzerland mail@kjablonka.com; b Department of Chemical Engineering, Massachusetts Institute of Technology Cambridge Massachusetts 02139 USA; c Department of Chemistry, McGill University Montreal Quebec Canada; d Reincarnate Inc. USA; e Yusuf Hamied Department of Chemistry, University of Cambridge Lensfield Road Cambridge CB2 1EW UK; f Laboratory of Artificial Chemical Intelligence (LIAC), Institut des Sciences et Ingénierie Chimiques, Ecole Polytechnique Fédérale de Lausanne (EPFL) Lausanne Switzerland; g National Centre of Competence in Research (NCCR) Catalysis, Ecole Polytechnique Fédérale de Lausanne (EPFL) Lausanne Switzerland; h Independent Researcher San Diego CA USA; i Mechanical Engineering and Materials Science, Duke University USA; j Material Measurement Laboratory, National Institute of Standards and Technology Maryland 20899 USA; k Department of Chemical Engineering, University of Rochester USA; l Applied Mathematics and Computational Research Division, Lawrence Berkeley National Laboratory Berkeley CA 94720 USA; m Institut de la Matière Condensée et des Nanosciences (IMCN), UCLouvain Chemin des Étoiles 8 Louvain-la-Neuve 1348 Belgium; n Matgenix SRL 185 Rue Armand Bury 6534 Gozée Belgium; o Instituto de Ciencia y Tecnología del Carbono (INCAR), CSIC Francisco Pintado Fe 26 33011 Oviedo Spain; p Department of Computer Science, University of Chicago Chicago Illinois 60637 USA; q Computer Science, University of California Berkeley CA 94704 USA; r Bundesanstalt für Materialforschung und -prüfung Unter den Eichen 87 12205 Berlin Germany; s Francis Crick Institute 1 Midland Rd London NW1 1AT UK; t American Family Insurance Data Science Institute, University of Wisconsin–Madison Madison WI 53706 USA; u Department of Chemical Engineering, University of Washington Seattle WA 98105 USA; v OpenBioML.org UK; w Stability.AI UK; x Department of Chemistry and Biochemistry, University of California Los Angeles CA 90095 USA; y Department of Computer Science, University of Chicago Chicago IL 60490 USA; z Data Science and Learning Division, Argonne National Lab USA; a Globus, University of Chicago, Data Science and Learning Division, Argonne National Lab USA blaiszik@uchicago.edu; b Department of Computer Science, University of Chicago, Data Science and Learning Division, Argonne National Lab USA

## Abstract

Large-language models (LLMs) such as GPT-4 caught the interest of many scientists. Recent studies suggested that these models could be useful in chemistry and materials science. To explore these possibilities, we organized a hackathon. This article chronicles the projects built as part of this hackathon. Participants employed LLMs for various applications, including predicting properties of molecules and materials, designing novel interfaces for tools, extracting knowledge from unstructured data, and developing new educational applications. The diverse topics and the fact that working prototypes could be generated in less than two days highlight that LLMs will profoundly impact the future of our fields. The rich collection of ideas and projects also indicates that the applications of LLMs are not limited to materials science and chemistry but offer potential benefits to a wide range of scientific disciplines.

## Abstract 2

We report the findings of a hackathon focused on exploring the diverse applications of large language models in molecular and materials science.

## Graphical abstract

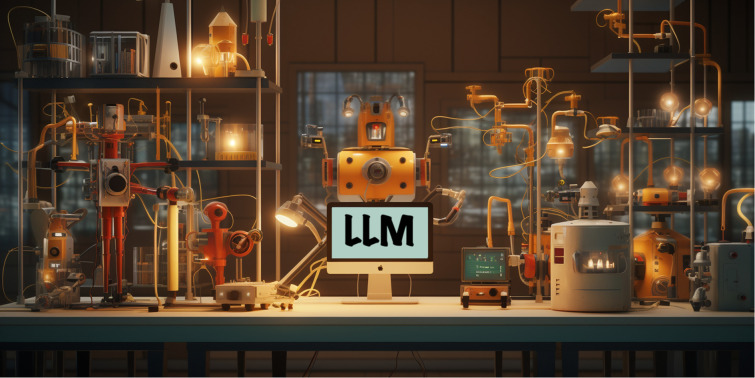

## 1. Introduction

The intersection of machine learning (ML) with chemistry and materials science has witnessed remarkable advancements in recent years.^1–9^ Much progress has been made in using ML to, *e.g.*, accelerate simulations^10,11^ or to directly predict properties or compounds for a given application.^12^ Thereby, developing custom, hand-crafted models for any given application is still common practice. Since science rewards doing novel things for the first time, we now face a deluge of tools and machine-learning models for various tasks. These tools commonly require input data in their own *rigid*, *well-defined form* (*e.g.*, a table with specific columns or images from a specific microscope with specific dimensions). Further, they typically also report their outputs in non-standard and sometimes proprietary forms.

This rigidity sharply contrasts the standard practice in the (experimental) molecular and materials sciences, which is intrinsically *fuzzy and highly context-dependent*.^13^ For instance, researchers have many ways to refer to a molecule (*e.g.*, IUPAC name, conventional name, simplified molecular-input line-entry system (SMILES)^14^) and to report results and procedures. In particular, for the latter, it is known that small details such as the order of addition or the strength of stirring (*e.g.*, “gently” *vs.* “strongly”) are crucial in determining the outcome of reactions. We do not have a natural way to deal with this fuzziness, and often a conversion into structured tabular form (the conventional input format for ML models) is impossible. Our current “solution” is to write conversion programs and chain many tools with plenty of application-specific “glue code” to enable scientific workflows. However, this fuzziness of chemistry and heterogeneity of tools have profound consequences: a never-ending stream of new file formats, interfaces, and interoperability tools exists, and users cannot keep up with learning.^15^ In addition, almost any transformation of highly context-dependent text (*e.g.*, description of a reaction procedure) into structured, tabular form will lead to a loss of information.

One of the aims of this work is to demonstrate how large language models (LLMs) such as the generative pretrained transformer (GPT)-4,^16–21^ can be used to address these challenges. Foundation models such as GPTs are general-purpose technologies^22^ that can solve tasks they have not explicitly been trained on,^23,24^ use tools,^25–27^ and be grounded in knowledge bases.^28,29^ As we also show in this work, they provide new pathways of exploration, new opportunities for flexible interfaces, and may be used to effectively solve certain tasks themselves; *e.g.*, we envision LLMs enabling non-experts to program (“malleable software”) using natural language as the “programming language”,^30^ extract structured information, and create digital assistants that make our tools interoperable—all based on unstructured, natural-language inputs.

Inspired by early reports on the use of these LLMs in chemical research,^31–34^ we organized a virtual hackathon event focused on understanding the applicability of LLMs to materials science and chemistry. The hackathon aimed to explore the multifaceted applications of LLMs in materials science and chemistry and encourage creative solutions to some of the pressing challenges in the field. This article showcases some of the projects (Table 1) developed during the hackathon.

**Table 1:** Overview of the developed tools and links to source code repositories. Full descriptions of the projects can be found in the ESI

Name	Authors	Links
**Predictive modeling**		
Accurate molecular energy predictions	Ankur K. Gupta, Garrett W. Merz, Alishba Imran, Wibe A. de Jong	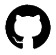
		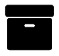 https://doi.org/10.5281/zenodo.8104930
Text2Concrete	Sabine Kruschwitz, Christoph Völker, Ghezal Ahmad Zia	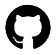 https://ghezalahmad/LLMs-for-the-Design-of-Sustainable-Concretes
		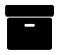 https://doi.org/10.5281/zenodo.8091195
Molecule discovery by context	Zhi Hong, Logan Ward	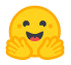 https://globuslabs/ScholarBERT-XL
		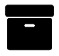 https://doi.org/10.5281/zenodo.8122087
Genetic algorithm without genes	Benjamin Weiser, Jerome Genzling, Nicolas Gastellu, Sylvester Zhang, Tao Liu, Alexander Al-Feghali, Nicolas Moitessier, Anne Labarre, Steven Ma	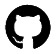 https://BenjaminWeiser/LLM-Guided-GA
		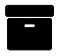 https://doi.org/10.5281/zenodo.8125541
Text-template paraphrasing	Michael Pieler	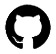 https://micpie/text-template-paraphrasing-chemistry
		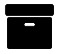 https://doi.org/10.5281/zenodo.8093615
**Automation and novel interfaces**		
BOLLaMa	Bojana Ranković, Andres M. Bran, Philippe Schwaller	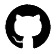 https://doncamilom/BOLLaMa
		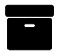 https://doi.org/10.5281/zenodo.8096827
sMolTalk	Jakub Lála, Sean Warren, Samuel G. Rodriques	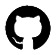 https://jakublala/smoltalk-legacy
		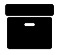 https://doi.org/10.5281/zenodo.8081749
MAPI-LLM	Mayk Caldas Ramos, Sam Cox, Andrew White	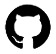 https://maykcaldas/MAPI_LLM
		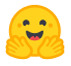 https://maykcaldasMAPI_LLM
		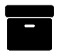 https://doi.org/10.5281/zenodo.8097336
Conversational electronic lab notebook (ELN) interface ( 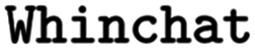 )	Joshua D. Bocarsly, Matthew L. Evans and Ben E. Smith	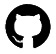 https://the-grey-group/datalab
		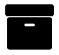 https://doi.org/10.5281/zenodo.8127782
**Knowledge extraction**		
InsightGraph	Defne Circi, Shruti Badhwar	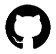 https://defnecirci/InsightGraph
		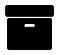 https://doi.org/10.5281/zenodo.8092575
Extracting structured data from free-form organic synthesis text	Qianxiang Ai, Jacob N. Sanders, Jiale Shi, Stefan Bringuier, Brenden Pelkie, Marcus Schwarting	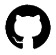 https://qai222LLM_organic_synthesis
		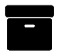 https://doi.org/10.5281/zenodo.8091902
TableToJson: structured information from scientific data in tables	María Victoria Gil	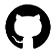 https://vgvinter/TableToJson
		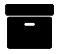 https://doi.org/10.5281/zenodo.8093731
AbstractToTitle & TitleToAbstract: text summarization and generation	Kamal Choudhary	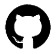 https://usnistgov/chemnlp
		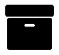 https://doi.org/10.5281/zenodo.8122419
**Education**		
I-Digest	Beatriz Mouriño, Elias Moubarak, Joren Van Herck, Sauradeep Majumdar, Xiaoqi Zhang	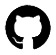 https://XiaoqZhang/i-Digest
		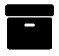 https://doi.org/10.5281/zenodo.8080962

One of the conclusions of this work is that without these LLMs, such projects would take many months. The diversity of topics these projects address illustrates the broad applicability of LLMs; the projects touch many different aspects of materials science and chemistry, from the wet lab to the computational chemistry lab, software interfaces, and even the classroom. While the examples below are not yet polished products, the simple observation that such capabilities could be created in hours underlines that we need to start thinking about how LLMs will impact the future of materials science, chemistry, and beyond.^35^ The diverse applications show that LLMs are here to stay and are likely a foundational capability that will be integrated into most aspects of the research process. Even so, the pace of the developments highlights that we are only beginning to scratch the surface of what LLMs can do for chemistry and materials science.

Table 1 lists the different projects created in this collaborative effort across eight countries and 22 institutions (ESI Section V†). One might expect that 1.5 days of intense collaborations would, at best, allow a cursory exploration of a topic. However, the diversity of topics and the diversity in the participants' expertise, combined with the need to deliver a working prototype (within a short window of time) and the ease of prototyping with LLMs, generated not only many questions but also pragmatic prototypes. The projects were typically carried out in an exploratory way and without any evaluation of impact. In the remainder of this article, we focus on the insights we obtained from this collective effort. For the details of each project, we refer to the ESI.† While different challenges were explored during this hackathon, the results were preliminary. *Digital Discovery* did not peer review the soundness of each study. Instead, the peer review for this perspective was to scope the potential of LLMs in chemistry and materials science.

We have grouped the projects into four categories: (1) predictive modeling, (2) automation and novel interfaces, (3) knowledge extraction, and (4) education. The projects in the *predictive modeling* category use LLMs for classification and regression tasks—and also investigate ways to incorporate established concepts such as Δ-ML^36^ or novel concepts such as “fuzzy” context into the modeling. The *automation and novel interfaces* projects show that natural language might be the universal “glue” connecting our tools—perhaps in the future, we will need not to focus on new formats or standards but rather use natural language descriptions to connect across the existing diversity and different modalities.^35^

LLMs can also help make knowledge more accessible, as the projects in the “knowledge extraction” category show; they can extract structured information from unstructured text. In addition, as the project in the “education” category shows, LLMs can also offer new educational opportunities.

### 1.1 Predictive modeling

Predictive modeling is a common application of ML in chemistry. Based on the language-interfaced fine-tuning (LIFT) framework,^37^ Jablonka *et al.*^32^ have shown that LLMs can be employed to predict various chemical properties, such as solubility or HOMO–LUMO gaps based on line representations of molecules such as self-referencing embedded strings (SELFIES)^38,39^ and SMILES. Taking this idea even further, Ramos *et al.*^34^ used this framework (with in-context learning (ICL)) for Bayesian optimization—guiding experiments without even training models. These few-shot learning abilities have also been benchmarked by Guo *et al.*^40^

The projects in the following build on top of those initial results and extend them in novel ways as well as by leveraging established techniques from quantum machine learning.

Given that these encouraging results could be achieved with and without fine-tuning (*i.e.*, updates to the weights of the model) for the language-interfaced training on tabular datasets, we use the term LIFT also for ICL settings in which structured data is converted into text prompts for an LLM.

#### 1.1.1 Molecular energy predictions

A critical property in quantum chemistry is the atomization energy of a molecule, which gives us the basic thermochemical data used to determine a molecule's stability or reactivity. State-of-the-art quantum chemical methods (*i.e.*, G4(MP2)^41^) can predict this energy with an accuracy of 0.034 eV (or 0.79 kcal mol^−1^).^42,43^ This accuracy is similar to, and in some cases even better than, the accuracy that can be reached experimentally. This motivated Ramakrishnan *et al.*^42^ and Narayanan *et al.*^43^ to compute these atomization energies for the 134 000 molecules in the QM9-G4MP2 dataset.

The Berkeley–Madison team (Ankur Gupta, Garrett Merz, Alishba Imran, and Wibe de Jong) used this dataset to fine-tune different LLMs using the LIFT framework. The team investigated if they could use an LLM to predict atomization energies with chemical accuracy. Jablonka *et al.*^32^ emphasized that these LLMs might be particularly useful in the low-data limit. Here, we have a relatively large dataset, so it is an ideal system to gather insights into the performance of these models for datasets much larger than those used by Jablonka *et al.*^32^

The Berkeley–Madison team showed that the LIFT framework based on simple line representations such as SMILES and SELFIES^38,39^ can yield good predictions (*R*^2^ > 0.95 on a holdout test set), that are, however, still inferior to dedicated models that have access to 3D information.^44,45^ An alternative approach to achieve chemical accuracy with LLMs tuned only on string representations is to leverage a Δ-ML scheme^46^ in which the LLM is tuned to predict the difference between G4(MP2) and B3LYP^47^ energies. Table 2 shows that good agreement could be achieved for the Δ-ML approach. This showcases how techniques established for conventional ML on molecules can also be applied with LLMs.

**Table 2:** LIFT for molecular atomization energies on the QM9-G4MP2 dataset. Metrics for models tuned on 90% of the QM9-G4MP2 dataset (117 232 molecules), using 10% (13 026 molecules) as a holdout test set. GPTChem refers to the approach reported by Jablonka *et al.*,^32^ GPT-2-LoRA to PEFT of the GPT-2 model using LoRA. The results indicate that the LIFT framework can also be used to build predictive models for atomization energies, that can reach chemical accuracy using a Δ-ML scheme. Baseline performance (mean absolute error reported by Ward *et al.*^45^): 0.0223 eV for FCHL-based prediction of GP4(MP2) atomization energies and 0.0045 eV (SchNet) and 0.0052 eV (FCHL) for the Δ-ML scheme

Mol. repr. & framework	G4(MP2) atomization energy		(G4(MP2)-B3LYP) atomization energy	
	*R* ^2^	Median absolute deviation (MAD)/eV	*R* ^2^	MAD/eV
SMILES: GPTChem	0.984	0.99	0.976	0.03
SELFIES: GPTChem	0.961	1.18	0.973	0.03
SMILES: GPT2-LoRA	0.931	2.03	0.910	0.06
SELFIES: GPT2-LoRA	0.959	1.93	0.915	0.06

Importantly, this approach is not limited to the OpenAI application programming interface (API). With parameter efficient fine-tuning (PEFT) with low-rank adaptors (LoRA)^48^ of the GPT-2 model,^49^ one can also obtain comparable results on consumer hardware. These results make the LIFT approach widely more accessible.

#### 1.1.2 Text2Concrete

Concrete is the most used construction material, and the mechanical properties and climate impact of these materials are a complex function of the processing and formulation. Much research is focused on formulations of concrete that are less CO_2_ intensive.^50^ To expedite the design process, *e.g.*, by prioritizing experiments using ML-predictions, data-driven methods have been investigated by Völker *et al.*^51^ The Text2Concrete team (Sabine Kruschwitz, Christoph Völker, and Ghezal Ahmad Zia) explored, based on data reported by Rao and Rao,^52^ whether LLMs can be used for this task. This data set provides 240 alternative, more sustainable, concrete formulations and their respective compressive strengths. From a practical point of view, one would like to have a model that can predict the compressive strength of the concrete as a function of its formulation.

Interestingly, the largest LLMs can already give predictions without any fine-tuning. These models can “learn” from the few examples provided by the user in the prompt. Of course, such a few-shot approach (or ICL,^20^) does not allow for the same type of optimization as fine-tuning, and one can therefore expect it to be less accurate. However, Ramos *et al.*^34^ showed that this method could perform well—especially if only so few data points are available such that fine-tuning is not a suitable approach.

**Fig. 1:**
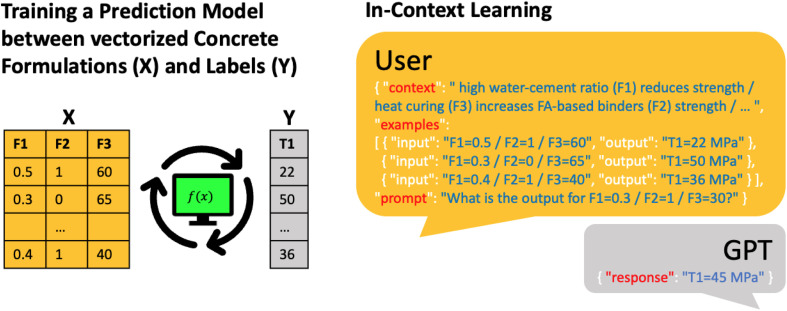
Using LLMs to predict the compressive strength of concretes. An illustration of the conventional approach for solving this task, *i.e.*, training classical prediction models using ten training data points as tabular data (left). Using the LIFT framework LLMs can also use tabular data and leverage context information provided in natural language (right). The context can be “fuzzy” design rules often known in chemistry and materials science but hard to incorporate in conventional ML models. Augmented with this context and ten training examples, ICL with LLM leads to a performance that outperforms baselines such as RFs or GPR.

For their case study, the Text2Concrete team found a predictive accuracy comparable to a Gaussian process regression (GPR) model (but inferior to a random forest (RF) model). However, one significant advantage of LLMs is that one can *easily incorporate context*. The Text2Concrete team used this to include well-established design principles like the influence of the water-to-cement ratio on strength (Fig. 1) into the modeling by simply stating the relationship between the features in natural language (*e.g.*, “high water/cement ratio reduces strength”). This additional context reduced the outliers and outperformed the RF model (*R*^2^ of 0.67 and 0.72, respectively).

The exciting aspect is that this is a typical example of domain knowledge that cannot be captured with a simple equation incorporable into conventional modeling workflows. Such “fuzzy” domain knowledge, which may sometimes exist only in the minds of researchers, is common in chemistry and materials science. With the incorporation of such “fuzzy” knowledge into LIFT-based predictions using LLMs, we now have a novel and very promising approach to leverage such domain expertise that we could not leverage before. Interestingly, this also may provide a way to test “fuzzy” hypotheses, *e.g.*, a researcher could describe the hypothesis in natural language and see how it affects the model accuracy. While the Text2Concrete example has not exhaustively analyzed how “fuzzy” context alterations affect LLM performance, we recognize this as a key area for future research.

#### 1.1.3 Molecule discovery by context

Much context is available in the full text of scientific articles. This has been exploited by Tshitoyan *et al.*^53^ who used a Word2Vec^54^ approach to embed words into a vector space. Word2Vec does so by tasking a model to predict for a word the probability for all possible next words in a vocabulary. In this way, word embeddings capture syntactic and semantic details of lexical items (*i.e.*, words). When applied to material science abstracts, the word embeddings of compounds such as Li_2_CuSb could be used for materials discovery by measuring their distance (cosine similarity) to concepts such as “thermoelectric”.^55^ However, traditional Word2Vec, as used by Tshitoyan *et al.*,^53^ only produces *static* embeddings, which remain unchanged after training. Word embeddings extracted from an LLM, on the other hand, are *contextualized* on the specific sequence (sentence) in which they are used and, therefore, can more effectively capture the contexts of words within a given corpus.^56^ Inspired by this, the GlobusLabs team (Zhi Hong, Logan Ward) investigated if similar embeddings could be used to discover hydrogen carrier molecules, that are relevant for energy storage applications. For this, they leverage the ScholarBert model^57^ trained on a large corpus of scientific articles collected by the public.resource.org nonprofit organization. For different candidate molecules, they searched for sentences in the public.resource.org corpus and used the average of the embeddings of these sentences as a fingerprint of the molecules. Given those fingerprints, they could rank molecules by how close their fingerprints are to the ones of known hydrogen carrier molecules. Visual inspection indicates that the selected molecules bear similarities to known hydrogen carrier molecules. Note that in this case, molecules are not generated *de novo* (as, for example, in Li *et al.*^58^) but retrieved from existing databases.

#### 1.1.4 Text template paraphrasing

In the LIFT framework used in the examples above, the data are embedded in so-called prompt templates that can have a form like 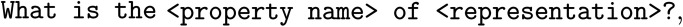 where the texts in chevrons are placeholders that are replaced with actual values such as “solubility” and “2-acetyloxybenzoic acid”. In the low-data regime, data points are “wasted” by the model needing to learn the syntax of the prompt templates. In the big-data regime, in contrast, one might worry that the model loses some of its general language modeling abilities by always dealing with the same template. This naturally raises the question if one can augment the dataset to mitigate these problems—thereby leveraging again, similar to Δ-ML, a technique that has found use in conventional ML previously. However, text-based data are challenging to augment due to their discrete nature and the fact that the augmented text still needs to be syntactically and semantically valid. Interestingly, as Michael Pieler (https://www.openbioml.org and Stability.AI) shows (and as has been explored by Dai *et al.*^59^), it turns out that LLMs can also be used to address this problem by simply prompting an LLM (*e.g.*, GPT-4 or Anthropic's Claude) to paraphrase a prompt template (see ESI Section ID†).

This approach will allow us to automatically create new paraphrased high-quality prompts for LIFT-based training very efficiently—to augment the dataset and reduce the risk of overfitting to a specific template. Latter might be particularly important if one still wants to retain general language abilities of the LLMs after finetuning on chemistry or material science data.

#### 1.1.5 Genetic algorithm using an LLM

Genetic algorithms are popular methods for generating new structures; they are evolutionary algorithms in which building blocks (*e.g.*, fragments of SMILES strings) are iteratively crossed over, mutated, and subjected to other genetic operations to evolve structures with better performance (such as catalysts with higher conversion).^60^ The efficiency of such a genetic algorithm often depends on how well the genes and genetic operations match the underlying chemistry. For example, if the algorithm replaces atom by atom, it may take several generations before a complete functional group is replaced.

One might hypothesize that LLMs can make the evolution process more efficient, *e.g.*, by using an LLM to handle the reproduction. One might expect that inductive biases in the LLM help create recombined molecules which are more chemically viable, maintaining the motifs of the two parent molecules better than a random operation.

**Fig. 2:**
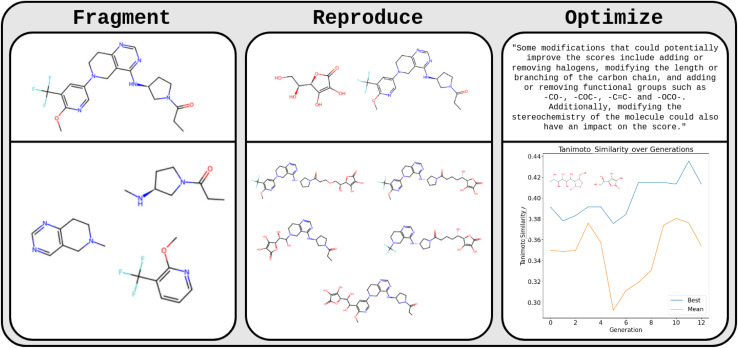
GA using an LLM. This figure illustrates how different aspects of a GA can be performed by an LLM. GPT-3.5 was used to fragment, reproduce, and optimize molecules represented by SMILES strings. The first column illustrated how an LLM can fragment a molecule represented by a SMILES string (input molecule on top, output LLM fragments below). The middle column showcases how an LLM can reproduce/mix two molecules as is done in a GA (input molecule on top, output LLM below). The right column illustrates an application in which an LLM is used to optimize molecules given their SMILES and an associated score. The LLM suggested potential modifications to optimize molecules. The plot shows best (blue) and mean (orange) Tanimoto similarity to vitamin C per LLM produced generations.

The team from McGill University (Benjamin Weiser, Jerome Genzling, Nicolas Gastellu, Sylvester Zhang, Tao Liu, Alexander Al-Feghali, Nicolas Moitessier) set out the first steps to test this hypothesis (Fig. 2). In initial experiments, they found that GPT-3.5, without any finetuning, can fragment molecules provided as SMILES at rotatable bonds with a success rate of 70%. This indicates that GPT-3.5 understands SMILES strings and aspects of their relation to the chemical structures they represent. Subsequently, they asked the LLMs to fragment and recombine two given molecules. The LLM frequently created new combined molecules with fragments of each species which were reasonable chemical structures more often than a random SMILES string combining operation (two independent organic chemists judged the LLM-GA-generated molecules to be chemically reasonable in 32/32 cases, but only in 21/32 cases for the random recombination operation).

Encouraged by these findings, they prompted an LLM with 30 parent molecules and their performance scores (Tanimoto similarity to vitamin C) with the task to come up with *n* new molecules that the LLM “believes” to improve the score. A preliminary visual inspection suggests that the LLM might produce chemically reasonable modifications. Future work will need to systematically investigate potential improvements compared to conventional GAs.

The importance of the results of the McGill team is that they indicate that these LLMs (when suitably conditioned) might not only reproduce known structures but generate new structures that make chemical sense.^32,61^

A current limitation of this approach is that most LLMs still struggle to output valid SMILES without explicit fine-tuning.^33^ We anticipate that this problem might be mitigated by building foundation models for chemistry (with more suitable tokenization^62,63^), as, for instance, the ChemNLP project of openbioml.org attempts to do (https://github.com/OpenBioML/chemnlp). In addition, the context length limits the number of parent molecules that can be provided as examples.

Overall, we see that the flexibility of the natural language input and the in-context learning abilities allows using LLMs in very different ways—to very efficiently build predictive models or to approach molecular and material design in entirely unprecedented ways, like by providing context—such as “fuzzy” design rules—or simply prompting the LLM to come up with new structures. However, we also find that some “old” ideas, such as Δ-ML and data augmentation, can also be applied in this new paradigm.

### 1.2 Automation and novel interfaces

Yao *et al.*^64^ and Schick *et al.*^25^ have shown that LLMs can be used as agents that can autonomously make use of external tools such as Web-APIs—a paradigm that some call MRKL (pronounced “miracle”) systems—modular reasoning, knowledge, and language systems.^26^ By giving LLMs access to tools and forcing them to think step-by-step,^65^ we can thereby convert LLMs from hyperconfident models that often hallucinate to systems that can reason based on observations made by querying robust tools. As the technical report for GPT-4 highlighted,^66^ giving LLMs access to tools can lead to emergent behavior, *i.e.*, enabling the system to do things that none of its parts could do before. In addition, this approach can make external tools more accessible—since users no longer have to learn tool-specific APIs. It can also make tools more interoperable—by using natural language instead of “glue code” to connect tools.

This paradigm has recently been used by Bran *et al.*^67^ to create digital assistants that can call and combine various tools such as Google search and the IBM RXN retrosynthesis tool when prompted with natural language. Boiko *et al.*^68^ used a similar approach and gave LLMs access to laboratories *via* cloud lab APIs. In their system, the LLM could use external tools to plan a synthesis, which it could execute using the cloud lab.

#### 1.2.1 MAPI-LLM

Electronic structure calculations have reached such a high level of accuracy that one can answer questions like “Is the material AnByCz stable?” Indeed, the Materials Project^69^ stores thermodynamic data on many components from which one can obtain a reasonable estimate of the stability of a given material. Or, if the material is not in the database, one can do a simulation instead. Similarly, to answer prompts such as “Give me a reaction to produce CaCO_3_”, there is a lot of helpful information in the Materials Project database and the internet that can help to come up with an answer.

To answer these questions, state-of-the-art computational tools or existing databases can be used. However, their use often requires expert knowledge. To use existing databases, one must choose which database to use, how to query the database, and what representation of the compound is used (*e.g.*, international chemical identifier (InChI), SMILES, *etc.*). Otherwise, if the data is not in a database, one must run calculations, which requires a deep understanding of technical details. LLMs can simplify this process. By typing in a question, we can prompt the LLM to translate this question into a workflow that leads to the answer.

The MAPI-LLM team (Mayk Caldas Ramos, Sam Cox, Andrew White) made the first steps towards developing such a system (MAPI-LLM) and created a procedure to convert a text prompt into a query of the Materials Project API (MAPI) to answer questions such as “Is the material AnByCz stable?” In addition, MAPI-LLM is capable of handling classification queries, such as “Is Fe_2_O_3_ magnetic?”, as well as regression problems, such as “What is the band gap of Mg(Fe_2_O_3_)_2_?”.

Because an LLM is used to create the workflow, MAPI-LLM can process even more complex questions. For instance, the question “If Mn_23_FeO_32_ is not metallic, what is its band gap?” should create a two-step workflow first to check if the material is metallic and then obtain its band gap if it is not.

**Fig. 3:**
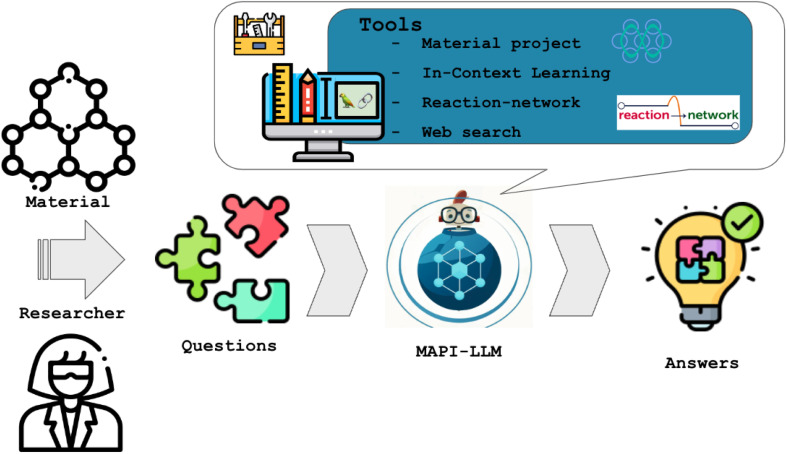
Schematic overview of the MAPI-LLM workflow. It uses LLMs to process the user's input and decide which available tools (*e.g.*, Materials Project API, the Reaction-Network package, and Google Search) to use following an iterative chain-of-thought procedure. In this way, it can answer questions such as “Is the material AnByCz stable?”.

Moreover, MAPI-LLM applies ICL if the data for a material's property is unavailable *via* the MAPI. MAPI-LLM generates an ICL prompt, building context based on the data for similar materials available in Materials Project database. This context is then leveraged by an LLM to infer properties for the unknown material. This innovative use of ICL bridges data gaps and enhances MAPI-LLM's robustness and versatility (Fig. 3).

#### 1.2.2 sMolTalk

The previous application already touches on the problem that software for chemical applications requires scientists to invest a significant amount of time in learning even the most basic applications. An example of this is visualization software. Depending on the package and its associated documentation, chemists and materials scientists might spend hours to days learning the details of specific visualization software that is sometimes poorly documented. And in particular, for occasional use, if it takes a long time to learn the basics, it won't be used.

**Fig. 4:**
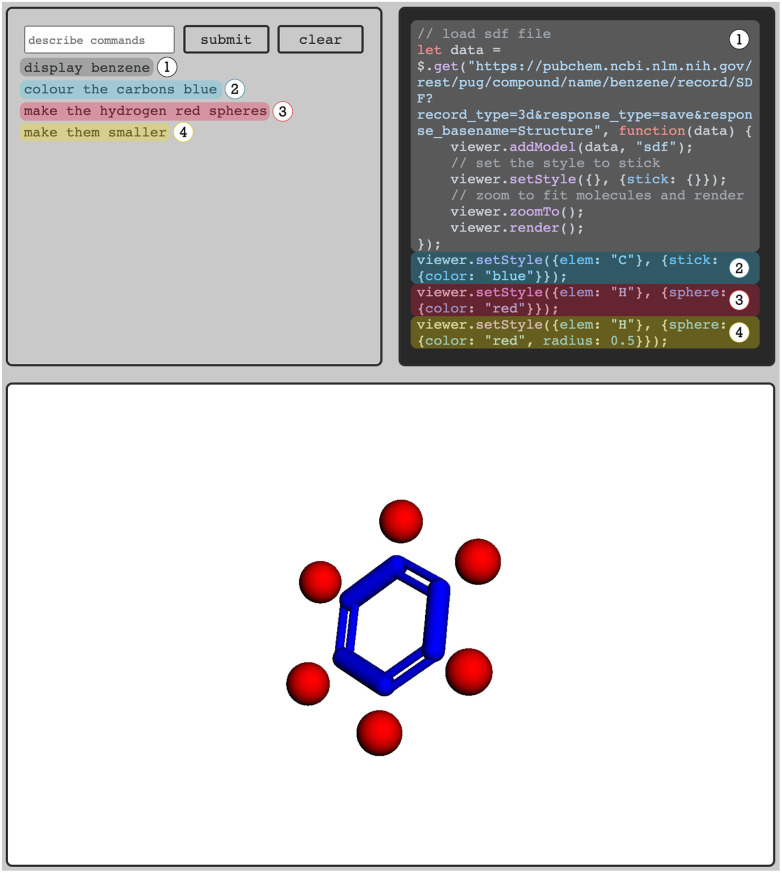
The sMolTalk interface. Based on few-shot prompting LLMs can create code for visualization tools such as 
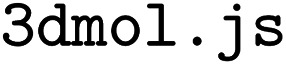
 that can create custom visualization based on a natural-language description of the desired output. The top left box is the input field where users can enter commands in natural language. The top right box prints the code the LLM generates. This code generates the visualization shown in the lower box. In this example, the user entered a sequence of four commands: the LLM (1) generates code for retrieving the structure, (2) colors the carbons blue, (3) displays the hydrogens as red spheres, and (4) reduces the size of the spheres.

As the sMolTalk-team (Jakub Lála, Sean Warren, Samuel G. Rodriques) showed, one can use LLMs to write code for visualization tools such as 
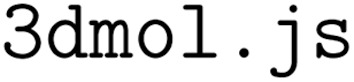
 to address this inefficiency.^70^ Interestingly, few-shot prompting with several examples of user input with the expected JavaScript code that manipulates the 
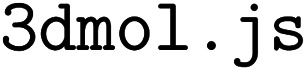
 viewer is all that is needed to create a prototype of an interface that can retrieve protein structures from the protein data bank (PDB) and create custom visualization solutions, *e.g.*, to color parts of a structure in a certain way (Fig. 4). The beauty of the language models is that the user can write the prompt in many different (“fuzzy”) ways: whether one writes “color” or “colour”, or terms like “light yellow” or “pale yellow” the LLM translates it into something the visualization software can interpret.

However, this application also highlights that further developments of these LLM-based tools are needed. For example, a challenge the sMolTalk tool faces is robustness. For instance, fragments from the prompt tend to leak into the output and must be handled with more involved mechanisms, such as retries (in which one gives the LLMs access to the error messages) or prompt engineering. Further improvement can also be expected if the application leverages a knowledge base such as the documentation of 
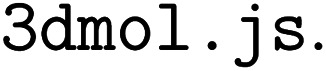


As the work of Hocky and White shows,^71^ an LLM-interface for software can also be used with other programs such as 
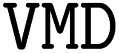
,^72^ and extended with speech-to-text models (such as Whisper^73^) to enable voice control of such programs. In particular, such an LLM-based agent approach might be implemented for the 
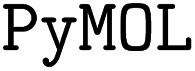
 program, where various tools for protein engineering could be interfaced through a chat interface, lowering the barrier to entry for biologists to use recent advancements within *in silico* protein engineering (such as RosettaFold^74^ or RFDiffusion^75^).

##### ELN interface: 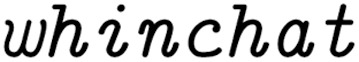


1.2.2.1

**Fig. 5:**
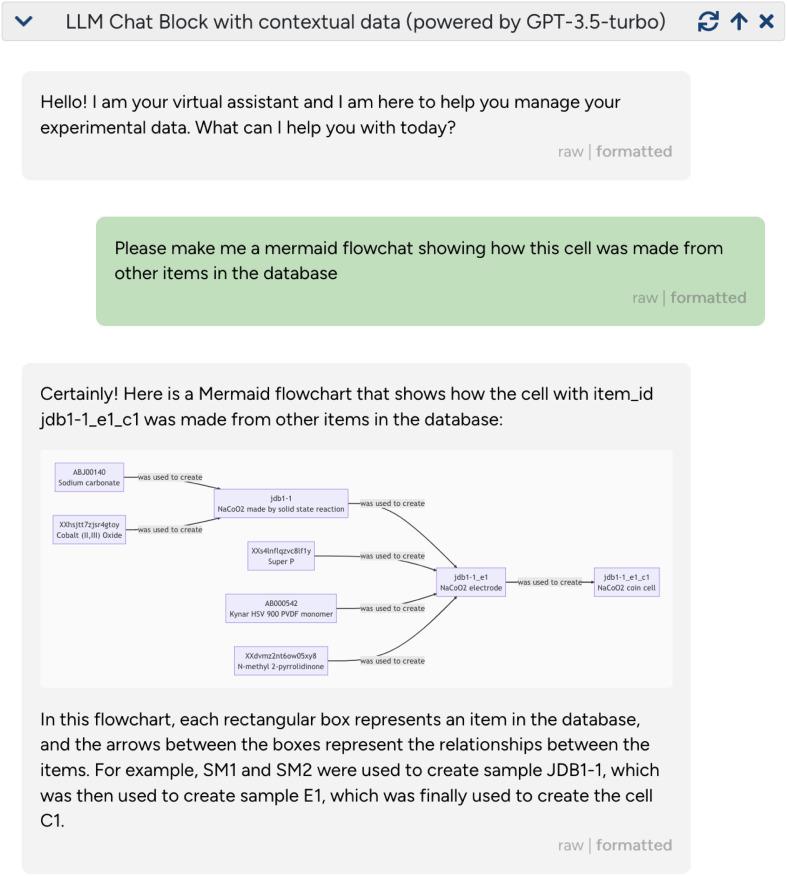
Using an LLM as an interface to an ELN/data management system/data management system. LLM-based assistants can provide powerful interfaces to digital experimental data. The figure shows a screenshot of a conversation with 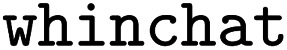 in the 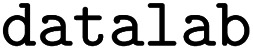 data management system (https://github.com/the-grey-group/datalab). Here, 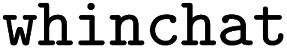 is provided with data from the JSON API of 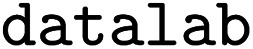 of an experimental battery cell. The user then prompts (green box) the system to build a flowchart of the provenance of the sample. The assistant responds with 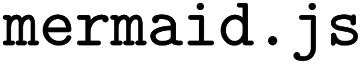 markdown code, which the 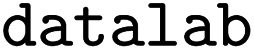 interface automatically recognizes and translates into a visualization.

In addition to large, highly curated databases with well-defined data models^76^ (such as those addressed by the MAPI-LLM project), experimental materials and chemistry data is increasingly being captured using digital tools such as ELNs and laboratory information systems (LIMS). Importantly, these tools can be used to record both structured and unstructured lab data in a manner that is actionable by both humans and computers. However, one challenge in developing these systems is that it is difficult for a traditional user interface to have enough flexibility to capture the richness and diversity of real, interconnected, experimental data. Interestingly, LLMs can interpret and contextualize both structured and unstructured data and can therefore be used to create a novel type of flexible, conversational interface to such experimental data. The 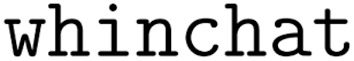 team (Joshua D. Bocarsly, Matthew L. Evans, and Ben E. Smith) embedded an LLM chat interface within 
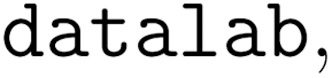
 an open source materials chemistry data management system, where the virtual LLM-powered assistant can be “attached” to a given sample. The virtual assistant has access to responses from the JavaScript object notation (JSON) API of 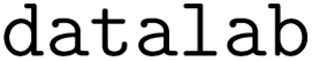 (containing both structured and unstructured/free text data) and can use them to perform several powerful tasks: first, it can contextualize existing data by explaining related experiments from linked responses, resolving acronyms/short-hand notations used by experimentalists, or creating concise textual summaries of complex and nested entries. Second, it can reformat or render the data, for instance, by creating (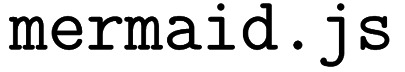) flowcharts or (Markdown) tables (Fig. 5). Third, it can use its generic reasoning abilities to suggest future experiments, for instance, related materials to study, synthesis protocols to try, or additional characterization techniques. This is shown in the examples given in ESI Section 2C,† where 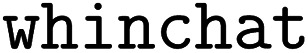 was able to provide hints about which NMR-active nuclei can be probed in the given sample.

It is easy to envision that this tool could be even more helpful by fine-tuning or conditioning it on a research group's knowledge base (*e.g.*, group Wiki or standard operating procedures) and communication history (*e.g.*, a group's Slack history). An important limitation of the current implementation is that the small context window of available LLMs limits the amount of JSON data one can directly provide within the prompt, limiting each conversation to analyzing a relatively small number of samples. Therefore, one needs to either investigate the use of embeddings to determine which samples to include in the context or adopt an “agent” approach where the assistant is allowed to query the API of the ELN (interleaved with extraction and summarization calls).

#### 1.2.3 BOLLaMa: facilitating Bayesian optimization with large language models

**Fig. 6:**
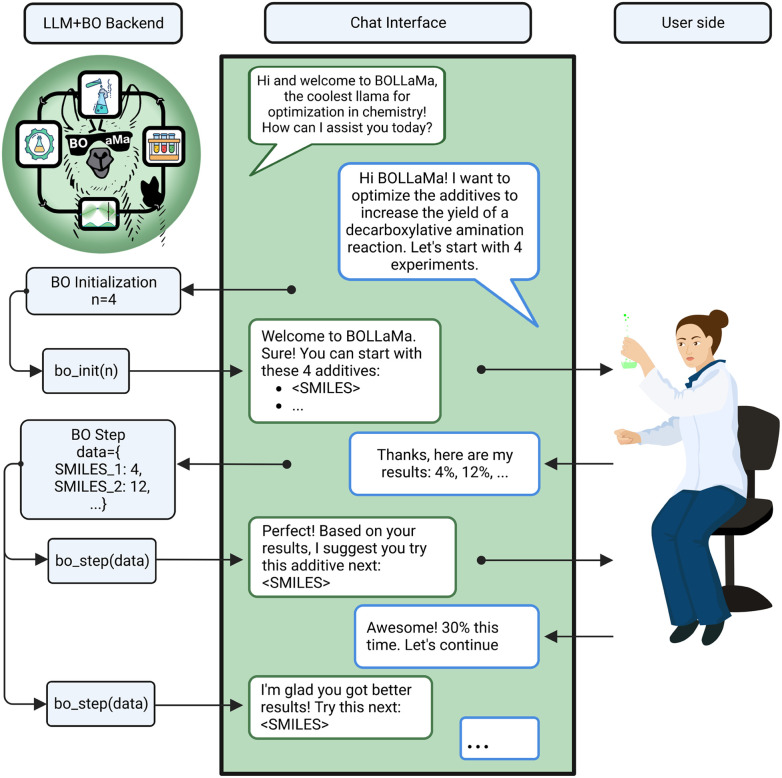
Schematic overview of BoLLama. An LLM can act as an interface to a BO algorithm. An experimental chemist can bootstrap an optimization and then, *via* a chat interface, update the state of the simulation to which the bot responds with the recommended next steps.

Bayesian optimization (BO) is a powerful tool for optimizing expensive functions, such as mapping of reaction conditions to the reaction yield. Chemists would greatly benefit from using this method to reduce the number of costly experiments they need to run.^77,78^ However, BO faces an interface and accessibility problem, too. The existing frameworks require significant background knowledge and coding experience not conventionally taught in chemistry curricula. Therefore, many chemists cannot benefit from tools such as BO. The BOLLaMa-team (Bojana Ranković, Andres M. Bran, Philippe Schwaller) showed that LLMs can lower the barrier for the use of BO by providing a natural language chat-like interface to BO algorithms. Fig. 6 shows a prototype of a chat interface in which the LLM interprets the user request, initializes a BO run by suggesting initial experimental conditions, and then uses the feedback of the user to drive the BO algorithm and suggest new experiments. The example used data on various additives for a cooperative nickel-photoredox catalyzed reaction^79^ and the BO code from Ranković *et al.*^80^ This ideally synergizes with an LLM interface to a data management solution (as discussed in the previous project) as one could directly persist the experimental results and leverage prior records to “bootstrap” BO runs.

As the examples in this section show, we find that LLMs have the potential to greatly enhance the efficiency of a diverse array of processes in chemistry and materials science by providing novel interfaces to tools or by completely automating their use. This can help streamline workflows, reduce human error, and increase productivity—often by replacing “glue code” with natural language or familiarising oneself with a software library by chatting with an LLM.

### 1.3 Knowledge extraction

Beyond proving novel interfaces for tools, LLMs can also serve as powerful tools for extracting knowledge from the vast amount of chemical literature. With LLMs, researchers can rapidly mine and analyze large volumes of data, enabling them to uncover novel insights and advance the frontiers of chemical knowledge. Tools such as paper-qa^28^ can help to dramatically cut down the time required for literature search by automatically retrieving, summarizing, and contextualizing relevant fragments from the entire corpus of the scientific literature—for example, answering questions (with suitable citations) based on a library of hundreds of documents.^35^ As the examples in the previous section indicated, this is particularly useful if the model is given access to search engines on the internet.

#### 1.3.1 InsightGraph

To facilitate downstream use of the information, LLMs can also convert unstructured data—the typical form of these literature reports—into structured data. The use of GPT for this application has been reported by Dunn *et al.*^81^ and Walker *et al.*,^82^ who used an iterative fine-tuning approach to extract data structured in JSON from papers. In their approach, initial (zero-shot) completions of the LLM are corrected by domain experts. Those corrected completions are then used to finetune LLMs, showing improved performance on this task.

**Fig. 7:**
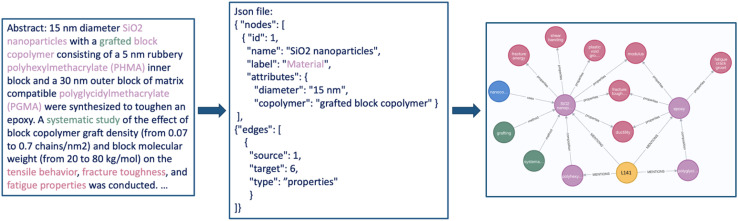
The InsightGraph interface. A suitably prompted LLM can create knowledge graph representations of scientific text that can be visualized using tools such as neo4j's visualization tools.^83^

However, for certain applications, one can construct powerful prototypes using only careful prompting. For instance, the InsightGraph team (Defne Circi, Shruti Badhwar) showed that GPT-3.5-turbo, when prompted with an example JSON containing a high-level schema and information on possible entities (*e.g.*, materials) and pairwise relationships (*e.g.*, properties of materials), can, as Fig. 7 illustrates, provide a knowledge graph representation of the entities and their relationships in a text describing the properties and composition of polymer nanocomposites. A further optimized version of this tool might offer a concise and visual means to understand and compare material types quickly and uses across sets of articles—a task that currently is very laborious. An advanced potential application is the creation of structured, materials-specific datasets for fact-based question-answering and downstream machine-learning tasks.

#### 1.3.2 Extracting structured data from free-form organic synthesis text

**Fig. 8:**
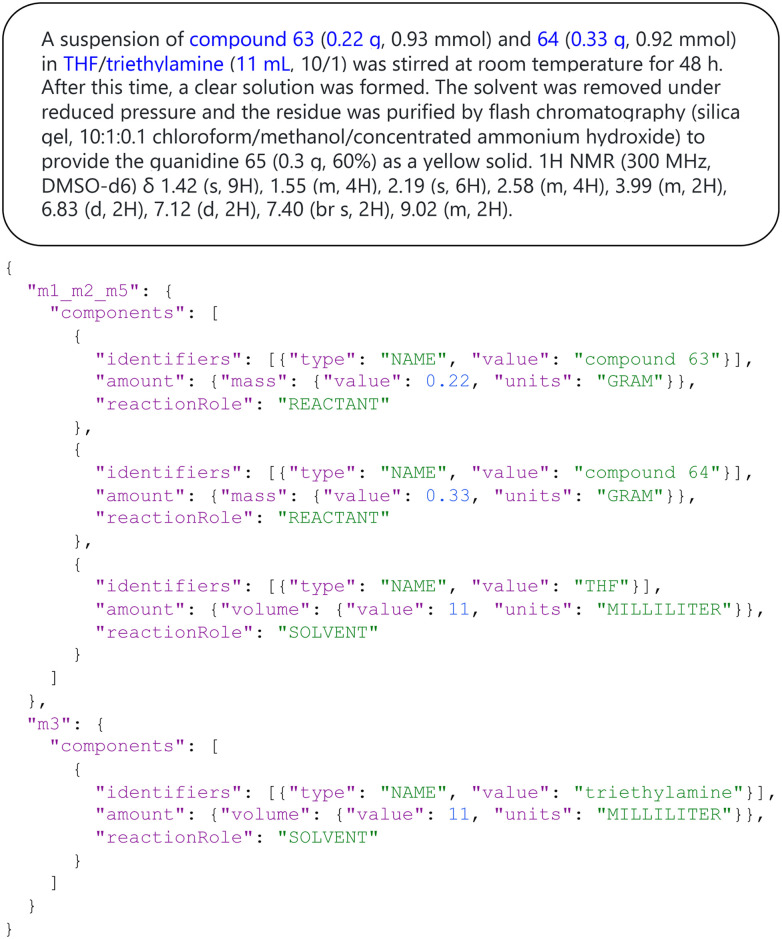
The organic synthesis parser interface. The top box shows text describing an organic reaction (https://open-reaction-database.org/client/id/ord-1f99b308e17340cb8e0e3080c270fd08), which the finetuned LLM converts into structured JSON (bottom). A demo application can be found at https://qai222.github.io/LLM_organic_synthesis/.

Unstructured text is commonly used for describing organic synthesis procedures. Due to the large corpus of literature, manual conversion from unstructured text to structured data is unrealistic. However, structured data are needed for building conventional ML models for reaction prediction and condition recommendation. The Open Reaction Database (ORD)^84^ is a database of curated organic reactions. In the ORD, while reaction data are structured by the ORD schema, many of their procedures are also available as plain text. Interestingly, an LLM (*e.g.*, OpenAI's 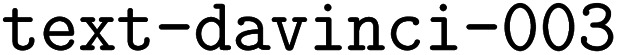) can, after finetuning on only 300 prompt–completion pairs, extract 93% of the components from the free-text reaction description into valid JSONs (Fig. 8). Such models might significantly increase the data available for training models on tasks such as predicting reaction conditions and yields. In contrast to previous approaches, such as the one of Guo *et al.*,^85^ the use of LLM does not require a specialized modeling setup but can be carried out with relatively little expertise. It is worth noting that all reaction data submitted to ORD are made available under the CC-BY-SA license, which makes ORD a suitable data source for fine-tuning or training an LLM to extract structured data from organic procedures. A recent study on gold nanorod growth procedures also demonstrated the ability of LLM in a similar task.^82^ In contrast to the LIFT-based prediction of atomization energies reported in the first section by the Berkeley–Madison team, parameter-efficient fine-tuning of the open-source Alpaca model^86–88^ using LoRA^48^ did not yield a model that can construct valid JSONs.

#### 1.3.3 TableToJson: structured information from tables in scientific papers

The previous example shows how structured data can be extracted from plain text using LLMs. However, relevant information in the scientific literature is not only found in text form. Research papers often contain tables that collect data on material properties, synthesis conditions, and results of characterization and experiments. Converting table information into structured formats is essential to enable automated data analysis, extraction, and integration into computational workflows. Although some techniques could help in the process of extracting this information (performing OCR or parsing XML), converting this information in structured data following, for example, a specific JSON schema with models remains a challenge. The INCAR-CSIC team (María Victoria Gil) showed that the OpenAI 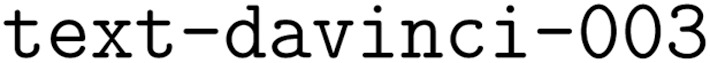 model, when prompted with a desired JSON schema and the HyperText Markup Language (HTML) of a table contained in a scientific paper, can generate structured JSON with the data in the table.

**Fig. 9:**
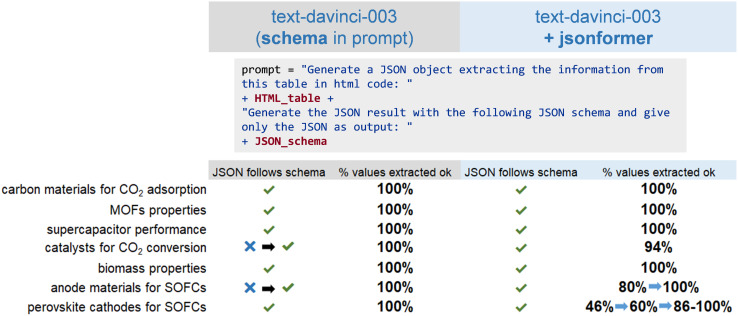
TableToJson. Results of the structured JSON generation of tables contained in scientific articles. Two approaches are compared: (i) the use of an OpenAI model prompted with the desired JSON schema, and (ii) the use of an OpenAI model together with 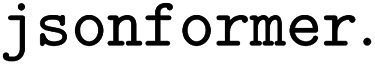 In both cases, JSON objects were always obtained. The output of the OpenAI model did not always follow the provided schema, although this might be solved by modifying the schema. The accuracy of the results from the 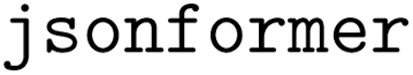 approach used with OpenAI models could be increased (as shown by the blue arrows) by solving errors in the generation of power numbers and special characters with a more detailed prompt. The results can be visualized in this demo app: https://vgvinter-tabletojson-app-kt5aiv.streamlit.app/.

First, the OpenAI 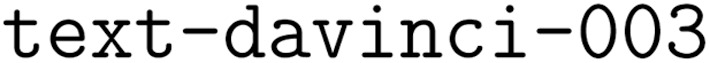 model was directly used to generate JSON objects from the table information. This approach was applied to several examples using tables collected from papers on different research topics within the field of chemistry.^89–95^ The accuracy for those different examples, calculated as the percentage of schema values generated correctly, is shown in Fig. 9. When the OpenAI model was prompted with the table and desired schema to generate a JSON object, it worked remarkably well in extracting the information from each table cell and inserting it at the expected place in the schema. As output, it provided a valid JSON object with a 100% success rate of error-free generated values in all the studied examples. However, in some examples, the model did not follow the schema.

To potentially address this problem the team utilized the 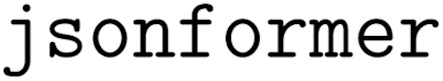 approach. This tool reads the keys from the JSON schema and only generates the value tokens, guaranteeing the generation of a syntactically valid JSON (corresponding to the desired schema) by the LLM.^96,97^ Using an LLM without such a decoding strategy cannot guarantee that valid JSON outputs are produced. With the 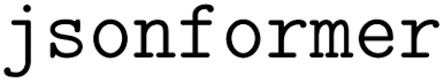 approach, in most cases, by using a simple descriptive prompt about the type of input text, structured data can be obtained with 100% correctness of the generated values. In one example, an accuracy of 80% was obtained due to errors in the generation of numbers in scientific notation. For a table with more complex content (long molecule names, hyphens, power numbers, subscripts, and superscripts,…) the team achieved an accuracy of only 46%. Most of these issues could be solved by adding a specific explanation in the prompt, increasing the accuracy to 100% in most cases.

Overall, both approaches performed well in generating the JSON format. The OpenAI 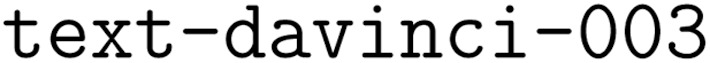 model could correctly extract structured information from tables and give a valid JSON output, but it cannot guarantee that the outputs will always follow the provided schema. 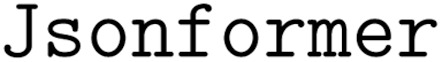 may present problems when special characters need to be generated, but most of these issues could be solved with careful prompting. These results show that LLMs can be a useful tool to help to extract scientific information in tables and convert it into a structured form with a fixed schema that can be stored in a database, which could encourage the creation of more topic-specific databases of research results.

#### 1.3.4 AbstractToTitle & TitleToAbstract: text summarization and text generation

Technical writing is a challenging task that often requires presenting complex abstract ideas in limited space. For this, frequent rewrites of sections are needed, in which LLMs could assist domain experts. Still, evaluating their ability to generate text such as a scientific paper is essential, especially for chemistry and materials science applications.

Large datasets of chemistry-related text are available from open-access platforms such as arXiv and PubChem. These articles contain titles, abstracts, and often complete manuscripts, which can be a testbed for evaluating LLMs as these titles and abstracts are usually written by expert researchers. Ideally, an LLM should be able to generate a title of an abstract close to the one developed by the expert, which can be considered a specialized text-summarization task. Similarly, given a title, an LLM should generate text close to the original abstract of the article, which can be considered a specialized text-generation task.

These tasks have been introduced by the AbstractToTitle & TitleToAbstract team (Kamal Choudhary) in the JARVIS-ChemNLP package.^98^ For text summarization, it uses a pre-trained Text-to-Text Transfer Transformer (T5) model developed by Google^99^ that is further fine-tuned to produce summaries of abstracts. On the arXiv condensed-matter physics (cond-mat) data, the team found that fine-tuning the model can help improve the performance (Recall-Oriented Understudy for Gisting Evaluation (ROUGE)-1 score of 39.0% which is better than an untrained model score of 30.8% for an 80/20 split).

For text generation, JARVIS-ChemNLP finetunes the pretrained GPT-2-medium^49^ model available in the HuggingFace library.^100^ After finetuning, the team found a ROUGE score of 31.7%, which is a good starting point for pre-suggestion text applications. Both tasks with well-defined train and test splits are now available in the JARVIS-Leaderboard platform for the AI community to compare other LLMs and systematically improve the performance.

In the future, such title to abstract capabilities can be extended to generating full-length drafts with appropriate tables, figures, and results as an initial start for the human researcher to help in the technical writing processes. Note that there have been recent developments in providing guidelines for using LLM-generated text in technical manuscripts,^101^ so such an LLM model should be considered as an assistant of writing and not the master/author of the manuscripts.

### 1.4 Education

Given all the opportunities LLM open for materials science and chemistry, there is an urgent need for education to adapt. Interestingly, LLMs also provide us with entirely novel educational opportunities,^102^ for example, by personalizing content or providing almost limitless varied examples.

**Fig. 10:**
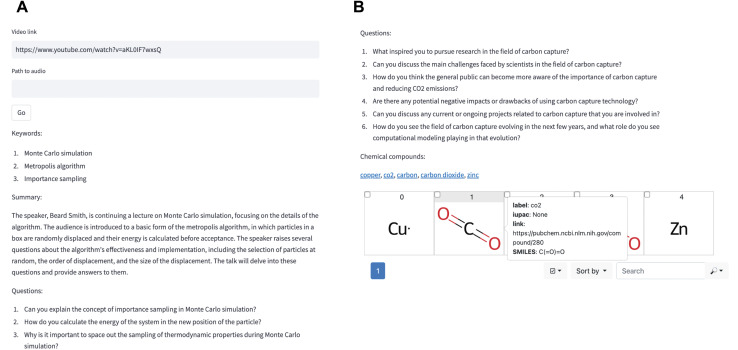
The I-digest interface. (a) A video (*e.g.*, of a lecture recording) can be described using the Whisper model. Based on the transcript, an LLM can generate questions (and answers). Those can assist students in their learning. (b) The LLM can also detect mentions of chemicals and link to further information about them (*e.g.*, on PubChem^103–105^).

The I-Digest (Information-Digestor) hackathon team (Beatriz Mouriño, Elias Moubarak, Joren Van Herck, Sauradeep Majumdar, Xiaoqi Zhang) created a path toward such a new educational opportunity by providing students with a digital tutor based on course material such as lecture recordings. Using the Whisper model,^73^ videos of lecture recordings can be transcribed to text transcripts. The transcripts can then be fed into an LLM with the prompt to come up with questions about the content presented in the video (Fig. 10). In the future, these questions might be shown to students before a video starts, allowing them to skip parts they already know or after the video, guiding students to the relevant timestamps or additional material in case of an incorrect answer.

Importantly, and in contrast to conventional educational materials, this approach can generate a practically infinite number of questions and could, in the future, continuously be improved by student feedback. In addition, it is easy to envision extending this approach to consider lecture notes or books to guide the students further or even recommend specific exercises.

## 2. Conclusion

The fact that the groups were able to present prototypes that could do quite complex tasks in such a short time illustrates the power of LLMs. Some of these prototypes would have taken many months of programming just a few months ago, but the fact that LLMs could reduce this time to a few hours is one of the primary reasons for the success of our hackathon. Combined with the time-constrained environment in teams (with practically zero cost of “failure”), we found more energy and motivation. The teams delivered more results than in most other hackathons we participated in.

Through the LIFT framework, one can use LLMs to address problems that could already be addressed with conventional approaches—but in a much more accessible way (using the same approach for different problems), while also reusing established concepts such as Δ-ML. At the same time, however, we can use LLMs to model chemistry and materials science in novel ways; for example, by incorporating context information such as “fuzzy” design rules or directly operating on unstructured data. Overall, a common use case has been to use LLMs to deal with “fuzziness” in programming and tool development. We can already see tools like Copilot and ChatGPT being used to convert “fuzzy abstractions” or hard-to-define tasks into code. These advancements may soon allow everyone to write small apps or customize them to their needs (end-user programming). Additionally, we can observe an interesting trend in tool development: most of the logic in the showcased tools is written in English, not in Python or another programming language. The resulting code is shorter, easier to understand, and has fewer dependencies because LLMs are adept at handling fuzziness that is difficult to address with conventional code. This suggests that we may not need more formats or standards for interoperability; instead, we can simply describe existing solutions in natural language to make them interoperable. Exploring this avenue further is exciting, but it is equally important to recognize the limitations of LLMs, as they currently have limited interpretability and lack robustness.

It is interesting to note that none of the projects relied on the knowledge or understanding of chemistry by LLMs. Instead, they relied on general reasoning abilities and provided chemistry information through the context or fine-tuning. However, this also brings new and unique challenges. All projects used the models provided by OpenAI's API. While these models are powerful, we cannot examine how they were built or have any guarantee of continued reliable access to them.

Although there are open-source language models and techniques available, they are generally more difficult to use compared to simply using OpenAI's API. Furthermore, the performance of language models can be fragile, especially for zero- or few-shot applications. To further investigate this, new benchmarks are needed that go beyond the tabular datasets we have been using for ML for molecular and materials science—we simply have no frameworks to compare and evaluate predictive models that use context, unstructured data, or tools. Without automated tests, however, it is difficult to improve these systems systematically. On top of that, consistent benchmarking is hard because de-duplication is ill-defined even if the training data are known. To enable a scientific approach to the development and analysis of these systems, we will also need to revisit versioning frameworks to ensure reproducibility as systems that use external tools depend on the exact versions of training data, LLM, as well as of the external tools and prompting setup.

The diversity of the prototypes presented in this work shows that the potential applications are almost unlimited, and we can probably only see the tip of the iceberg—for instance, we didn't even touch modalities other than text thus far. In addition, we also want to note that the projects in the workshop mostly explored the use of LLMs as tools or oracles but not as muses.^106^ From techniques such as rubber duck debugging (describing the problem to a rubber duck),^107^ we know that even simple—non-intelligent—articulation or feedback mechanisms can help overcome roadblocks and create creative breakthroughs. Instead of explaining a problem to an inanimate rubber duck, we could instead have a conversation with an LLM, which could probe our thinking with questions or aid in brainstorming by generating diverse new ideas. Therefore, one should expect an LLM to be as good as a rubber duck—if not drastically more effective.

Given these new ways of working and thinking, combined with the rapid pace of developments in the field, we believe that we urgently need to rethink how we work and teach. We must discuss how we ensure safe use,^108^ standards for evaluating and sharing those models, and robust and reliable deployments. But we also need to discuss how we ensure that the next generation of chemists and materials scientists are proficient and critical users of these tools—that can use them to work more efficiently while critically reflecting on the outputs of the systems. This work showcased some potential applications of LLMs that will benefit from further investigation. We believe that to truly leverage the power of LLMs in the molecular and material sciences, however, we need a community effort—including not only chemists and computer scientists but also lawyers, philosophers, and ethicists: the possibilities and challenges are too broad and profound to tackle alone.

## Data availability

The code and data for the case studies reported in this article can be found in the GitHub repositories linked in Table 1.

## Conflicts of interest

There are no conflicts to declare.

## Supplementary Material

DD-002-D3DD00113J-s001
