# Supplementary material for: 14 examples of how LLMs can transform materials science and chemistry: a reflection on a large language model hackathon
Source: Digit Discov. 2023 Aug 8;2(5):1233–50. doi: 10.1039/d3dd00113j (PMC10561547; doi:10.1039/d3dd00113j)
Supplement: DD-002-D3DD00113J-s001 [file DD-002-D3DD00113J-s001.pdf]

Electronic Supplementary Material (ESI) for Digital Discovery.
This journal is © The Royal Society of Chemistry 2023

Supporting Information for: 14 Examples of How LLMs Can Transform Materials Science and Chemistry: A Reflection on a Large Language Model Hackathon

Kevin Maik Jablonka [1,*] Qianxiang Ai [2,†] Alexander Al-Feghali [3,†] Shruti Badhwar [4,†] Joshua D. Bocarsly [5,†] Andres M Bran [6,7,†] Stefan Bringuier [8,†] L. Catherine Brinson [9,†] Kamal Choudhary [10,†] Defne Circi [9,†] Sam Cox [11,†] Wibe A. de Jong [12,†] Matthew L. Evans [13,14,†] Nicolas Gastellu [3,†] Jerome Genzling [3,†] María Victoria Gil [15,†] Ankur K. Gupta [12,†] Zhi Hong [16,†] Alishba Imran,[17,†] Sabine Kruschwitz [18,†] Anne Labarre [3,†] Jakub Lála [19,†] Tao Liu [3,†] Steven Ma [3,†] Sauradeep Majumdar [1,†] Garrett W. Merz [20,†] Nicolas Moitessier [3,†] Elias Moubarak [1,†] Beatriz Mouriño [1,†] Brenden Pelkie [21,†] Michael Pieler [22,23,†] Mayk Caldas Ramos [11,†] Bojana Ranković [6,7,†] Samuel G. Rodriques [19,†] Jacob N. Sanders [24,†] Philippe Schwaller [6,7,†] Marcus Schwarting,[25,†] Jiale Shi [2,†] Berend Smit [1,†] Ben E. Smith [5,†] Joren Van Herck [1,†] Christoph Völker [18,†] Logan Ward [26,†] Sean Warren [3,†] Benjamin Weiser [3,†] Sylvester Zhang,[3,†] Xiaoqi Zhang [1,†] Ghezal Ahmad Zia [18,†] Aristana Scourtas [27] KJ Schmidt,[27] Ian Foster [28] Andrew D. White [11] and Ben Blaiszik [27,‡]

[1] *Laboratory of Molecular Simulation (LSMO), Institut des Sciences et Ingénierie Chimiques, Ecole Polytechnique Fédérale de Lausanne (EPFL), Sion, Valais, Switzerland.*
[2] *Department of Chemical Engineering, Massachusetts Institute of Technology, Cambridge, Massachusetts 02139, United States.*
[3] *Department of Chemistry, McGill University, Montreal, Quebec, Canada.*
[4] *Reincarnate Inc.*
[5] *Yusuf Hamied Department of Chemistry, University of Cambridge, Lensfield Road, Cambridge, CB2 1EW, United Kingdom.*
[6] *Laboratory of Artificial Chemical Intelligence (LIAC), Institut des Sciences et Ingénierie Chimiques, Ecole Polytechnique Fédérale de Lausanne (EPFL), Lausanne, Switzerland.*
[7] *National Centre of Competence in Research (NCCR) Catalysis, Ecole Polytechnique Fédérale de Lausanne (EPFL), Lausanne, Switzerland.*
[8] *Independent Researcher, San Diego, CA, United States.*
[9] *Mechanical Engineering and Materials Science, Duke University, United States.*
[10] *Material Measurement Laboratory, National Institute of Standards and Technology, Maryland, 20899, United States.*
[11] *Department of Chemical Engineering, University of Rochester, United States.*
[12] *Applied Mathematics and Computational Research Division, Lawrence Berkeley National Laboratory, Berkeley, CA 94720, United States.*
[13] *Institut de la Matière Condensée et des Nanosciences (IMCN), UCLouvain, Chemin des Étoiles 8, Louvain-la-Neuve, 1348, Belgium.*
[14] *Matgenix SRL, 185 Rue Armand Bury, 6534 Gozée, Belgium.*
[15] *Instituto de Ciencia y Tecnología del Carbono (INCAR), CSIC, Francisco Pintado Fe 26, 33011 Oviedo, Spain.*
[16] *Department of Computer Science, University of Chicago, Chicago, Illinois 60637, United States.*
[17] *Computer Science, University of California, Berkeley, Berkeley CA 94704, United States.*
[18] *Bundesanstalt für Materialforschung und -prüfung, Unter den Eichen 87, 12205 Berlin, Germany.*
[19] *Francis Crick Institute, 1 Midland Rd, London NW1 1AT, United Kingdom.*
[20] *American Family Insurance Data Science Institute, University of Wisconsin-Madison, Madison WI 53706, United States.*
[21] *Department of Chemical Engineering, University of Washington, Seattle, WA 98105, United States.*
[22] *OpenBioML.org*
[23] *Stability.AI*
[24] *Department of Chemistry and Biochemistry, University of California, Los Angeles, CA 90095, United States.*
[25] *Department of Computer Science, University of Chicago, Chicago IL 60490, United States.*
[26] *Data Science and Learning Division, Argonne National Lab, United States.*
[27] *Globus, University of Chicago, Data Science and Learning Division, Argonne National Lab, United States.*

[28]*Department of Computer Science, University of Chicago, Data Science and Learning Division, Argonne National Lab, United States.*

Contents

I. Predictive Modeling 3
A. Leveraging LLMs for Accurate Molecular Energy Predictions 3
B. From Text to Cement: Developing Sustainable Concretes Using In-Context Learning 6
C. Molecule Discovery by Context 8
D. Text template paraphrasing with LLMs 10
1. Problem 10
2. Solution 10
3. Impact 12
4. Lessons learned 12
E. GA without genes 13

II. Automation and novel interfaces 18
A. Using chain-of-thought and chemical tools to answer materials questions 18
B. sMolTalk 20
C. `whinchat`: A Conversational electronic lab notebook (ELN) Interface 22
D. BOLLaMa 25

III. Knowledge Extraction 27
A. InsightGraph 27
B. Extracting Structured Data from Free-form Organic Synthesis Text 29
C. TableToJson: Extracting structured information from tables in scientific papers 31
D. AbstractToTitle & TitleToAbstract: text summarization and text generation 36
1. Problem 36
2. Solution 36
3. Example 36

IV. Education 38
A. i-Digest 38

V. Meta analysis of the workshop contributions 40

References 42

* mail@kjablonka.com
† These authors contributed equally
‡ blaiszik@uchicago.edu

I. Predictive Modeling

A. Leveraging LLMs for Accurate Molecular Energy Predictions

Table I. *LIFT for molecular atomization energies on the QM9-G4MP2 dataset.* Metrics for models tuned on 90% of the QM9-G4MP2 dataset (117,232 molecules), using 10% (13,026 molecules) as a holdout test set. Note that the metric used for the baseline results [1] is MAE, whereas this work used the MAD. The results indicate that the LIFT framework can also be used to build predictive models for atomization energies, that can reach chemical accuracy using a Δ-ML scheme.

mol. repr. & framework	G4(MP2) Atomization Energy		(G4(MP2)-B3LYP) Atomization Energy	
	R^2	MAD / eV	R^2	MAD / eV
SMILES: GPTChem	0.984	0.99	0.976	0.03
SELFIES: GPTChem	0.961	1.18	0.973	0.03
SMILES: GPT2-LoRA	0.931	2.03	0.910	0.06
SELFIES: GPT2-LoRA	0.959	1.93	0.915	0.06
SchNet baseline	-	-	-	0.0045
FCHL baseline	-	0.0223	-	0.0052

Accurate prediction of chemical properties has long been the ultimate objective in computational chemistry and materials science. However, the significant computational demands of precise methods often hinder their routine application in modeling chemical processes. The recent surge in machine learning development, along with the subsequent popularity of large language models (LLMs), offers innovative and effective approaches to overcome these computational limitations. Our project takes steps toward establishing a comprehensive, open-source framework that harnesses the full potential of LLMs to accurately model chemical problems and uncover novel solutions to chemical challenges. In this study, we assessed the capability of LLMs to predict the atomization energies of molecules at the G4(MP2) [2] level of theory from the QM9-G4MP2 dataset [3, 4] using solely string representations for molecules, specifically, SMILES [5] and SELFIES [6, 7]. G4(MP2) is a highly accurate composite quantum chemistry method, known for its accuracy within 1.0 kcal/mol for molecular energies compared to experimental values, making atomization energy an ideal property to predict to demonstrate the usefulness and impact of LLMs on the field of computational chemistry.

Jablonka et al. [8] recently demonstrated the potential of fine-tuning pre-trained LLMs on chemistry datasets for a broad array of predictive chemistry tasks. As an initial validation for our project, we fine-tuned generative pretrained transformer (GPT)-3 [9] to learn how to reproduce a molecule's atomization energy at the G4(MP2) level of theory, using its SMILES or SELFIES string through the prompt, "What is the G4MP2 atomization energy in kcal/mol of 'SMILES/SELFIES string of a molecule'?" Additionally, we fine-tuned LLMs to predict the atomization energy difference between B3LYP/6-31G(2df,p) and G4(MP2) levels of theory with the prompt, "What is the G4MP2 and B3LYP atomization energy difference in kcal/mol of 'SMILES/SELFIES string of a molecule'?", which mirrors the Δ-machine learning (Δ-ML) schemes [10] found in the existing literature.

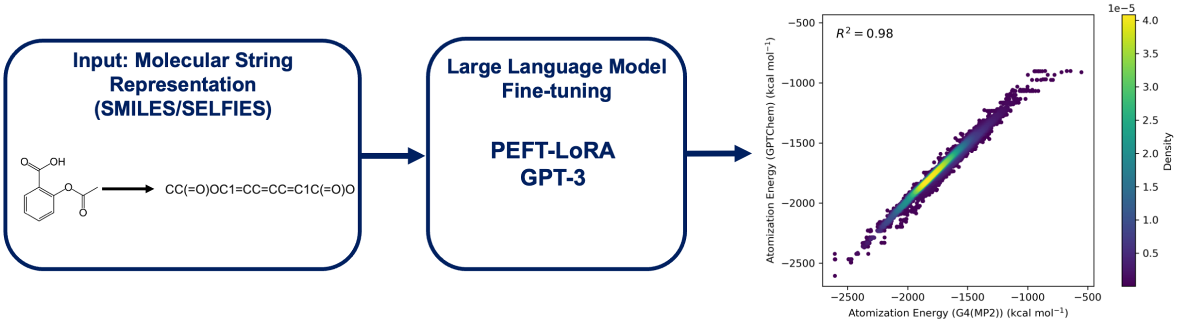

Figure 1. *Illustration of the molecular property prediction workflow, from molecule representation to model fine-tuning and performance evaluation.*

We fine-tuned the GPT-3 (Ada) model using 90% of the QM9-G4MP2 dataset (117,232 molecules) for eight epochs with the GPTChem [8] framework's default settings. The remaining 10 % (13,026 molecules) was kept as the hold-out set, following the same data split as Ward et al. [1], to evaluate the model's performance. Table I summarizes the regression metrics for the hold-out set. The strong correlation between the predicted and ground truth values suggests that the model effectively learned the structural information from the molecular string representation. Although the MAD remains relatively high compared to state-of-the-art models in the literature [1, 11] that utilize a molecule's full 3D structural information for descriptor construction, we achieved chemical accuracy ($< 1.0\,\mathrm{kcal/mol} \approx 0.04\,\mathrm{eV}$) for the Δ-ML task. Consequently, this approach can predict G4(MP2) energies with high accuracy when B3LYP energies are available. We also compared the model's performance using SMILES and SELFIES molecular representations, with the former proving marginally superior for predicting atomization energies, possibly due to its more compact representation for molecules. We additionally calculated regression metrics for the G4MP2-Heavy dataset [1], the results of which are provided in Table II.

Table II. Regression metrics, (Coefficient of Determination), and MAD (Mean Absolute Deviation) for predicting G4(MP2) and (G4(MP2)B3LYP) atomization energies for the G4MP2-Heavy dataset using a fine-tuned GPT-3 model with GPTChem

mol. repr. & framework	G4(MP2) Atomization Energy		(G4(MP2)-B3LYP) Atomization Energy	
	R^2	MAD / eV	R^2	MAD / eV
SMILES: GPTChem	0.583	6.02	0.856	0.13
SELFIES: GPTChem	0.146	9.44	0.659	0.15

While GPT-3 fine-tuning models are accessible through the OpenAI application programming interface (API), their usage costs can become prohibitive for larger datasets, rendering hyperparameter searches and other exploratory research economically unfeasible. Consequently, we aim to develop a free and open-source framework for fine-tuning LLMs to perform a wide range of predictive modeling tasks, encompassing chemical property prediction and inverse design.

To fine-tune a pre-trained LLM locally on a GPU instead of querying OpenAI's API, we employed the Hugging Face parameter efficient fine-tuning (PEFT) library [12] to implement the low-rank adaptors (LoRA) tuning paradigm [13]. Conventional fine-tuning updates all model parameters, utilizing pretrained weights from a large training dataset as a starting point for gradient descent. However, fine-tuning memory-intensive LLMs on consumer hardware is often impractical. The LoRA approach addresses this by freezing the model's weights and tuning a low-rank adapter layer rather than the entire model, parameterizing changes concerning the initial weights rather than the updated weights.

Using this approach, we fine-tuned the smallest version of GPT-2 [14] (124 million parameters) for 20 epochs on the same 90 % training set as used in GPTChem, allocating 10 % of that training set for validation, and computed metrics on the same 10 % hold-out set as in the GPTChem run, employing the same prompt structure. Although the model performs well, it demonstrates slightly inferior performance to GPT-3 on the G4MP2 task and moderately worse on the (G4(MP2)-B3LYP) task. This is not unexpected, given that GPT-3 is a more recent model with substantially more parameters than GPT-2 (175 billion vs. 124 million) and has exhibited superior few-shot performance on various tasks [15].

Moving forward, we plan to employ the LoRA tuning framework to fine-tune other models, such as LLaMA [16] and GPT-J, to investigate the impact of LLM selection on performance in chemistry-related tasks. Moreover, we intend to experiment with molecular-input representations beyond string formats to more accurately represent a molecule's 3D environment [17].

One sentence summaries

a. Problem/Task Predicting the atomization energies of molecules using large language models.

b. Approach Fine-tuning of GPT-3 `ada` model as well as PEFT of a small open-source model (GPT-2) on SMILES to either directly predict the atomization energies or the difference between a lower and a higher level of theory.

c. Results and Impact Even though simpler, direct fine-tuning for a complicated property on SMILES leads to errors one order of magnitude higher than baselines, and the error can only be brought close to the baselines with an $\Delta - ML$ approach—first demonstration of Δ-ML in the LIFT framework for chemistry.

d. Challenges and Future Work Since the predictions without 3D coordinates is not satisfactory, a question for future work is how the approach would perform when provided with 3D coordinates.

B. From Text to Cement: Developing Sustainable Concretes Using In-Context Learning

The inherently intricate chemistry and variability of feedstocks in the construction industry have limited the development of novel sustainable concretes to labor-intensive laboratory testing. This major bottleneck in material innovation has significant consequences due to the substantial contribution of CO_2 emissions of materials in use today. The production of Portland cement alone amounts to approximately 8 % of anthropogenic CO_2 emissions [18]. The increasing complexity of alternative raw materials and the uncertain future availability of established substitutes like fly ash and granulated blast furnace slag make the experimental development of more sustainable formulations time-consuming and challenging. Traditional trial-and-error approaches are ill-suited to efficiently explore the vast design space of potential formulations.

In previous studies, inverse design (ID) has been shown to accelerate the discovery of novel, sustainable, and high-performance materials by reducing labor-intensive laboratory testing [19–21]. Despite their potential, the adoption of these techniques has been impeded by several difficulties that are connected to the predictive model at the core of ID: Incorporating domain knowledge typically requires extensive data collection to accurately capture underlying relationships, which makes representing complex tasks in practice challenging due to the high costs of data acquisition. Furthermore, ID necessitates formulating research problems as search space vectors. This process can be unintuitive and challenging for lab personnel, limiting the comprehension and adoption of these techniques. Lastly, sparse training samples in high dimensions can lead to co-linearities and overfitting, negatively impacting prediction performance. With in-context learning

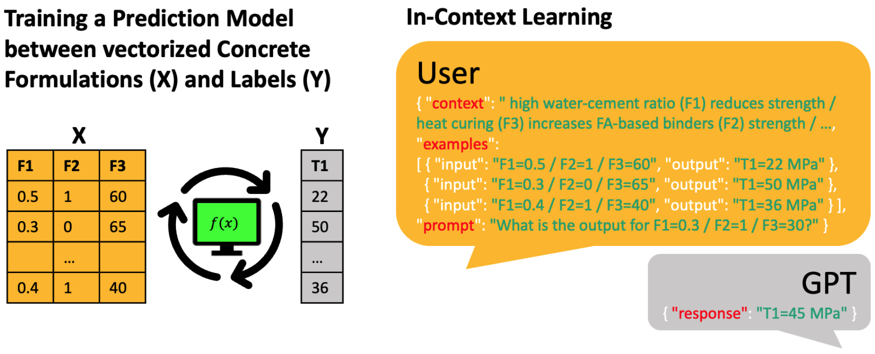

Figure 2. *Using LLMs to predict the compressive strength of concretes.* The left part illustrates the conventional approach for solving this task, i.e., training classical prediction models using tabular data. Using the LIFT framework LLM can also use tabular data but also leverage context information provided in natural language. Augmented with this context, in-context-learning with LLM leads to a performance that outperforms baselines such as RFs or GPRs.

(ICL), Jablonka et al. [8] and Ramos et al. [22] demonstrated that LLMs offer a solution by incorporating context and general knowledge, providing flexibility in handling non-numeric inputs and overcoming the limitations of traditional vector space formulations (Figure 2).

In this study, we have adopted an ICL approach based on a dataset from a study by Rao and Rao [23]. The dataset comprises 240 alternative and more sustainable concrete formulations based on fly ash and ground granulated slag binders, along with their respective compressive strengths. The goal is to compare the prediction performance of the compressive strength with ICL using the `text-davinci-003` model [24] against established methods, RF [25].

Randomly sampled training subsets containing ten formulations are drawn. The prediction performance is assessed on a separate, randomly sampled test set of 25 samples and evaluated using the coefficient of determination (R-squared) [26]. This process is repeated ten times to ensure more reliable results.

The experimental results reveal that ICL attains comparable performance to GPR but underperforms RF when provided with small training data sets (R-squared of 0.5, 0.54, and 0.67, respectively). However, when using general, qualitative concrete design knowledge, such as the influence of the water-to-cement ratio on strength, the models significantly reduce prediction outliers and ultimately surpass RF (R-squared = 0.71). When we incorrectly changed the context of the ratio of fly ash to GGBFS, it negatively affected the R-squared value for ICL, causing it to drop to 0.6. This misrepresentation of the rule led to a decrease in the model's predictive accuracy, demonstrating that the quality of the information included in the "fuzzy" context is critical to the overall performance of LLMs. It should be noted, however, that the impact on the R-squared value may vary depending on the importance of the rule in the overall context. That is, not all

changes in context have a similar impact, and the drop to 0.6 might occur only in the case of the ratio of fly ash to GGBFS. Other studies, such as those conducted in the LIFT work, [27] have shown LLM performance for minor changes in wording or the presence of noise in the features. In these experiments, the robustness of LIFT-based predictions was comparable to classical ML algorithms, making it a promising alternative for using fuzzy domain knowledge in predictive modeling.

LLMs have been shown to provide significant advantages in sustainable concrete development, including context incorporation, adaptable handling of non-numeric inputs, and efficient domain knowledge integration, surpassing traditional methods' limitations. ICLs simplifies formulating data-driven research questions, increasing accessibility and democratizing a data-driven approach within the building materials sector. This highlights LLMs potential to contribute to the construction industry's sustainability objectives and foster efficient solutions.

One sentence summaries

a. Problem/Task Predicting the compressive strength of concrete formulations.

b. Approach ICL on language-interfaced tabular data, with and without "fuzzy" domain expertise (such as relationship between columns) provided in natural language.

c. Results and Impact Predictive models can be built without any training (i.e., update of weights); if provided with domain expertise, those models outperform the baselines—first demonstration in chemistry of such fuzzy knowledge can be incorporated into models.

d. Challenges and Future Work ICL can be very sensitive to the prompt, hence future work should investigate the robustness of this approach.

C. Molecule Discovery by Context

The escalating climate crisis necessitates the deployment of clean, sustainable fuels to reduce carbon emissions. Hydrogen, with its potential to prevent approximately 60 gigatons of CO_2 emissions by 2050, according to the World Economic Forum, stands as a promising solution [28]. However, its storage and shipping remain formidable challenges due to the necessity for high-pressure tanks. To address this, we sought new molecules to which hydrogen could be conveniently added for storage. Traditional screening methods, like brainstorming, are insufficient due to their limited throughput. This research proposes a novel method of leveraging ScholarBERT, [29] a pre-trained science-focused LLM, to screen potential hydrogen carrier molecules efficiently. This approach utilizes ScholarBERT's ability to understand and relate the context of scientific literature. The data used for this study consisted of three datasets. The "Known" dataset comprised 78 known hydrogen carrier molecules. The "Relevant" dataset included 577 molecules, all of which are structurally similar to the "Known" molecules. The "Random" dataset contained 111 randomly selected molecules from the PubChem database [30]. The first step involved searching for contexts for molecules in the Public Resource Dataset (PRD), which includes 75M English language research articles. These contexts (i.e. sentences that mentioned the molecule name) were then fed into ScholarBERT. For each context, three calculations were made:

1. the average of the last four encoder layers in ScholarBERT
2. the average embedding of all tokens constituting the molecule name as one contextualized embedding for this molecule, and
3. the average of all contextualized embeddings for a molecule as ScholarBERT's representation of this molecule.

Subsequently, we calculated the similarity between the known and candidate molecules. The definition of "similarity" used in this study was the cosine similarity between the ScholarBERT representations of two molecules. We then sorted the candidates based on the similarity score in descending order, with a higher score indicating greater potential as a hydrogen carrier. Figure 3 and 4 show the candidate molecules with the highest similarity to the known molecules. We can see that ScholarBERT does a passable job finding similar molecules from the random set. We do see that it favors finding molecules with 5- and 6-member rings, though with features we didn't expect, like halogens. On the other hand, ScholarBERT does a much better job when we reduce the search space to those with structural similarity. We see that molecules with 5-member rings, for instance, are found to be similar structurally and in how they are described in the literature via ScholarBERT.

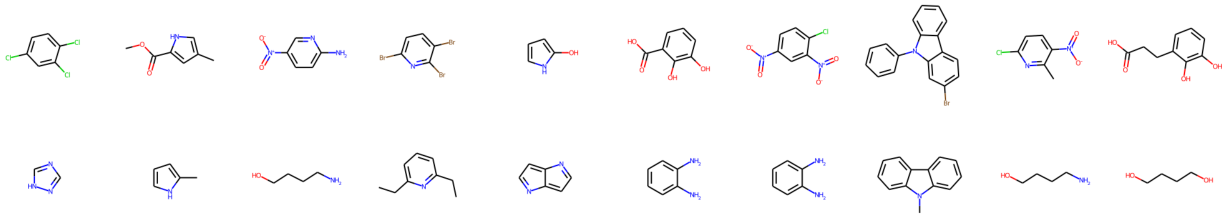

Figure 3. Each column shows a Known molecule on the bottom and its top candidate molecule from the Random set on the top

Based on our empirical data, computing the energy capacity (wt%H_2) and energy penalty (kJ/mol/H_2) of adding and removing H_2 to the molecule (which are the quantitative "success metrics" for this project) of a candidate molecule using traditional quantum chemistry takes around 30 seconds per molecule on a 64-core Intel Xeon Phi 7230 processor, whereas the proposed LLM approach can screen around 100 molecules per second on a V100 GPU, achieving a 3000 times speedup.

One sentence summaries

a. Problem/Task Recommending hydrogen carrier molecules.

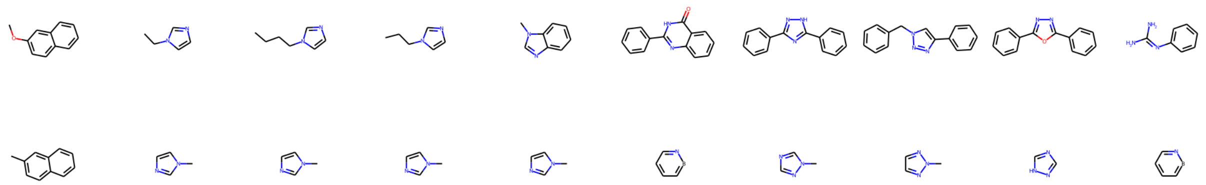

Figure 4. Each column shows a Known molecule on the bottom and its top candidate molecule from the Relevant set on the top

b. Approach Ranking of molecules based on similarity of embeddings created with a language model by aggregating the embeddings of mentions of the molecules in scientific texts.

c. Results and Impact Approach can recommend molecules with a success rate better than random.

d. Challenges and Future Work Since no direct comparisons to other approaches have been performed, benchmarks compared to conventional generative modeling are needed.

D. Text template paraphrasing with LLMs

1. *Problem*

Text data is much trickier to augment for machine learning applications due to the discrete nature of the data modality. There are some traditional augmentation approaches for these tasks. However, they can be inefficient or still need extensive manual checks to be sure they deliver the desired results, especially for scientific or chemistry applications.

2. *Solution*

To automate high-quality text data augmentations, LLMs have been explored by Dai and his coworkers [31] as a very recent and promising solution to this problem. We investigated such a setup in the scope of the OpenBioML chemistry NLP project (`https://github.com/OpenBioML/chemnlp`) to paraphrase text templates for the insertion of chemical raw data into natural language for LIFT. [27]

An example prompt is shown below. The outlined prompt setup has after "Question:" the desired task with additional information and after "Sentence:" the starting text template for the paraphrasing. The "Question:" and "Answer:" headers are not used if the LLM interface uses a chat interface, i.e., with OpenAI GPT-4.

Example Prompt

Question: Please paraphrase the sentence below ten times without changing the original meaning and the placeholder in the curly {} brackets. Please use all the placeholders in the curly {} brackets for every rephrased sentence.

Sentence: The molecule with the {SMILES_description} representation {SMILES_data} has a {target_name} of {target_value}{target_units}.

Answer:

Example Response by GPT-4

1. The {SMILES_description} representation of the molecule is {SMILES_data}, and its {target_name} is {target_value}{target_units}.
2. With a {SMILES_description} representation of {SMILES_data}, the molecule has a {target_name} of {target_value}{target_units}.
3. The molecule, represented by {SMILES_description} as {SMILES_data}, possesses a {target_name} of {target_value}{target_units}.
4. A molecule having a {SMILES_description} representation of {SMILES_data} also has a {target_name} equal to {target_value}{target_units}.
5. The {SMILES_description}-represented molecule with {SMILES_data} exhibits a {target_name} of {target_value}{target_units}.
6. For the molecule with a {SMILES_description} representation of {SMILES_data}, its {target_name} measures {target_value}{target_units}.
7. The {target_name} of the molecule having a {SMILES_description} representation of {SMILES_data} is {target_value}{target_units}.
8. Represented by {SMILES_description} as {SMILES_data}, the molecule's {target_name} is {target_value}{target_units}.

9. The molecule's {target_name} is {target_value}{target_units} when its {SMILES_description} representation is {SMILES_data}.
10. A {target_name} of {target_value}{target_units} is associated with the molecule whose {SMILES_description} representation is {SMILES_data}.

Example Response by Anthropic Claude v1

Here are 10 paraphrased sentences of the original sentence with the placeholders retained:

1. The molecule represented by the {SMILES_description} code {SMILES_data} has a {target_name} of {target_value}{target_units}.
2. The {SMILES_description} notation {SMILES_data} denotes a molecule with a {target_name} equal to {target_value}{target_units}.
3. A {target_name} of {target_value}{target_units} is possessed by the molecule depicted using {SMILES_description} as {SMILES_data}.
4. {SMILES_data} signifies the {SMILES_description} of the molecule which has a {target_name} of {target_value}{target_units}.
5. The {SMILES_description} {SMILES_data} characterizes the molecule that exhibits a {target_name} of {target_value}{target_units}.
6. A {target_name} equal to {target_value}{target_units} belongs to the molecule expressed with {SMILES_description} code {SMILES_data}.
7. {SMILES_data} denotes the {SMILES_description} for the molecule possessing a {target_name} of {target_value}{target_units}.
8. The molecule defined by {SMILES_description} {SMILES_data} presents a {target_name} of {target_value}{target_units}.
9. {SMILES_description} {SMILES_data} specifies the molecule which displays a {target_name} of {target_value}{target_units}.
10. A {target_name} amounting to {target_value}{target_units} is a property of the molecule represented with {SMILES_description} {SMILES_data}.

Example Response by GPT-J

The molecule with the {SMILES_description} representation {SMILES_A} has a {target_name} of {target_value}{target_units}. {SMILES_A} has a {target_name} of {target_units}. The molecule with the {target_units} representation {target_name} has a TARGET_UNITS of {target_value}{target_units}. {target_name} of {target_units} have a TARGET_UNITS of {target_value}{target_units}.

In the above answer, there is the {SMILES_description} representation of {SMILES_query}, but we don't use it in the sentence yet. And there is no curly brackets for the excepted answer.

3. Impact

The outlined approach allows to automatically create new paraphrased high-quality prompts for LIFT LLM training data very efficiently. With the additional paraphrased text templates, overfitting to special text passages should be avoided. We explore this setup in follow-up work in more detail.

4. Lessons learned

The outlined paraphrasing setup works well for the latest state-of-the-art models, e.g., OpenAI's GPT-4 and Anthropic's Claude v1. Less capable open-source models seem to lack the understanding of this paraphrasing task. Still, new and upcoming open-source LLM efforts could change that soon, enabling a cost-effective and broader application of this setup.

One sentence summaries

a. Problem/Task Generation of many text-templates for language-interfaced fine-tuning of LLMs

b. Approach Prompting of LLM to rephrase templates (with template syntax similar to Jinja).

c. Results and Impact Large models (GPT-4, Claude), in contrast to smaller ones, can successfully rephrase templates, offering a potential avenue for data-augmentation.

d. Challenges and Future Work As next step, ablation studies need to carried out that test the effect of data augmentation by template rephrasing on regression and classification case studies.

E. GA without genes

We investigate the ability for a LLM to work in parallel with genetic algorithms (GAs) for molecular property optimization. By employing a LLM to guide genetic algorithm operations, it could be possible to produce better results using fewer generations. We hypothesize that a GA can take advantage of the "smart" randomness of the outputs of the LLM. This work explores the potential of LLMs to improve molecular fragmentation, mutation, variation, and reproduction processes and the ability of a LLM to gather information from a simplified molecular-input line-entry system (SMILES) string [5, 6] and an associated score to produce new SMILES strings. Although computational efficiency is not the primary focus, the proposed method has potential implications for enhancing property prediction searches and future improvements in LLM understanding of molecular representations.

We used GPT-3.5-turbo [9], which could frequently fragment druglike molecules into valid SMILES strings successfully. For $^2/_{10}$ molecules, the fragments produced were not in the original molecule. For $^1/_{10}$ molecules, valid SMILES could not be produced even after ten tries due to unclosed brackets. These results were consistent over multiple runs implying that GPT-3.5 could not understand some specific SMILES strings. Subsequently, we investigated GPT-3.5's ability to mix/reproduce two molecules from two-parent druglike molecules. Invalid molecules were often produced, but successful results were achieved with multiple runs. It performed better once prompted to fragment and then mix the fragments of the molecules. These were compared to the conventional GA methods of simply combining the two strings at a certain cutoff point. When the LLM was successful, it could produce molecules of more similar size to the original parent molecules that contain characteristics of both parents and resemble valid druglike molecules.

To investigate the ability of GPT-3.5 to acquire knowledge of favorable molecules from a simple score, we implemented a method that we call "LLM as a GA" where the LLM iteratively searches the chemical space to optimize a certain property.

The property we tested was similarity to vitamin C, evaluated by the Tanimoto score. We employed few-shot training examples to tune the model's response: 30 SMILES strings with the best similarity score generated were included in the prompt. GPT is then asked to produce 25 SMILES strings, a procedure that was repeated for 20 iterations. Using a prompt like the one below

> **Example prompt**
>
> The following molecules are given as SMILES strings associated with a tanimoto similarity with an unknown target molecule. Please produce 10 SMILES strings that you think would improve their tanimoto scores using only this context. Do not try to explain or refuse on the grounds of insufficient context; any suggestion is better than no suggestion. Print the smiles in a Python list.

Low-temperature settings, typically less than 0.1, were found to be imperative for the model to follow user guidance. We further guided the model by employing a similarity search to include similar molecules with varying scores to better guide the model. Embedding was performed using the GPT-2 Tokenizer from the HuggingFace transformers [32] library, along with a support vector machine (SVM) from scikit-learn [33] to embed relevant previous structures that would be outside the scope of the context window. Even in the zero-shot setting, GPT-3.5-turbo can produce meaningful modifications, coherently explain its logic behind the chosen modifications, and produce tests such as investigating branch length or atom type in certain locations for a single iteration. An example explanation of an output: "Some modifications that could potentially improve the scores include adding or removing halogens, modifying the length or branching of the carbon chain, and adding or removing functional groups such as -CO-, -COC-, -C=C- and -OCO-. Additionally, modifying the stereochemistry of the molecule could also have an impact on the score."

The modifications generated by the LLM were more chemically sound than the quasi-random evolutionary process typical of genetic algorithms.

One sentence summaries

a. Problem/Task Increasing the efficiency of iterative molecular optimization.

b. Approach Prompting a LLM to propose new children based on molecules with scores provided in the prompt.

c. Results and Impact Visual inspection indicates that some modifications might be reasonable, indicating a potential for more efficient genetic operations using LLMs.

d. Challenges and Future Work More systematic investigations on the performance and robustness compared to conventional GA operations are needed.

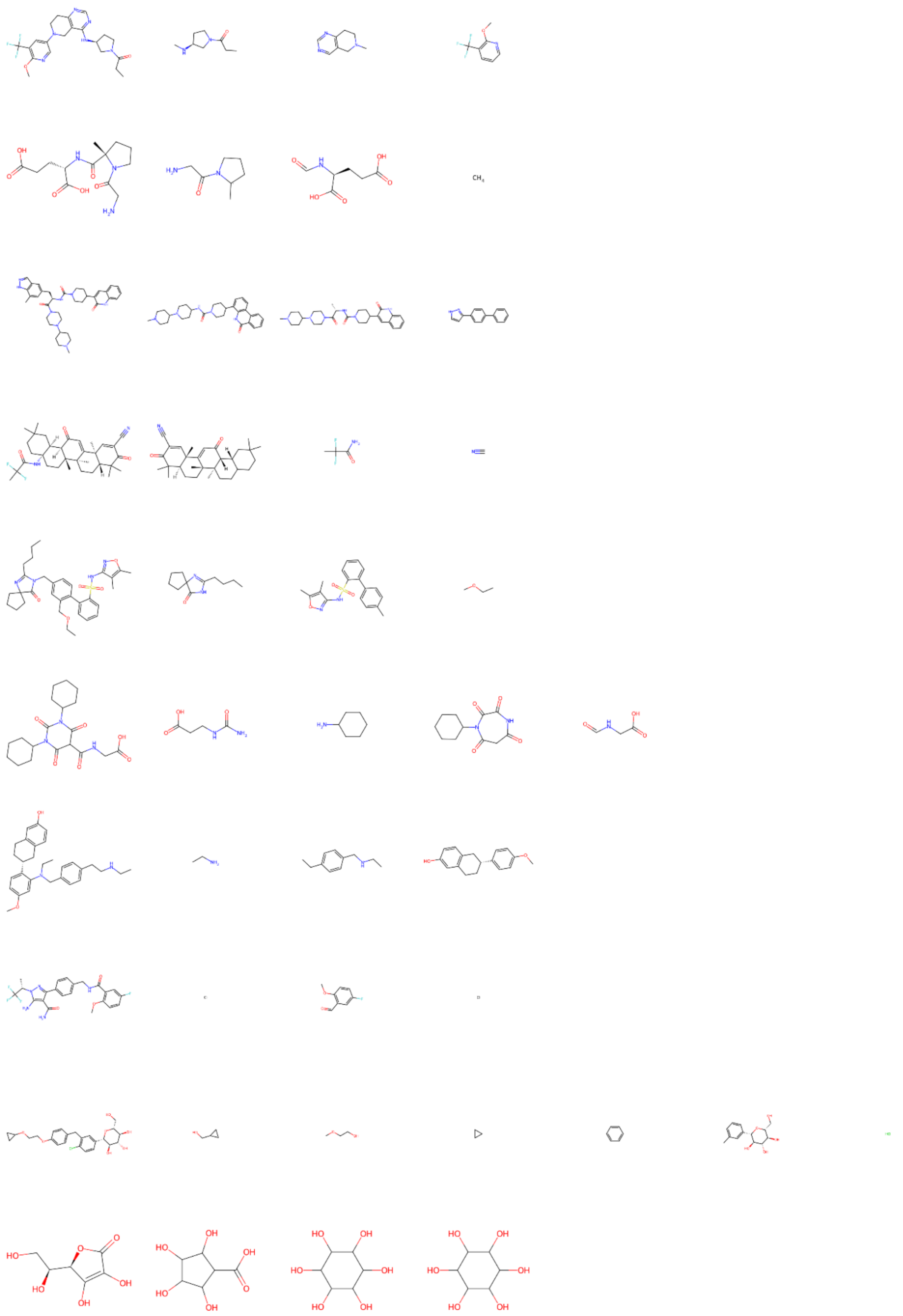

Figure 5. Using GPT to fragment molecules. Original molecules are in column one with LLM created fragment to the right. The LLM can frequently fragment molecules into valid SMILES strings successfully. 2/10 times fragments produced were not in the original molecule (rows 6 and 10). For 1/10 molecules, valid SMILES were able to be produced even after ten attempts (row 8)

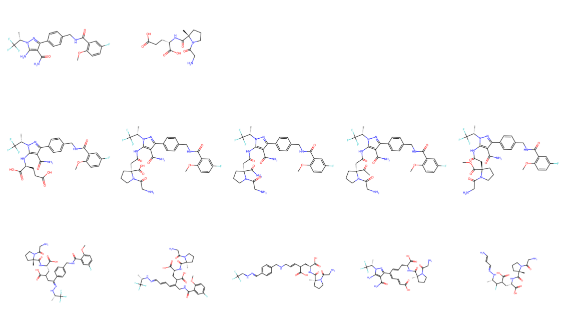

(a)

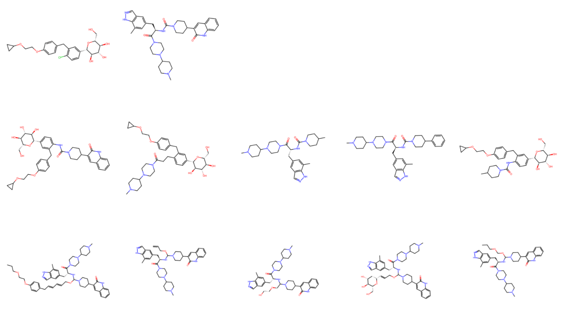

(b)

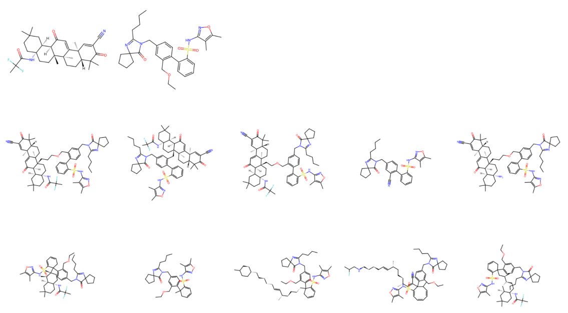

(c)

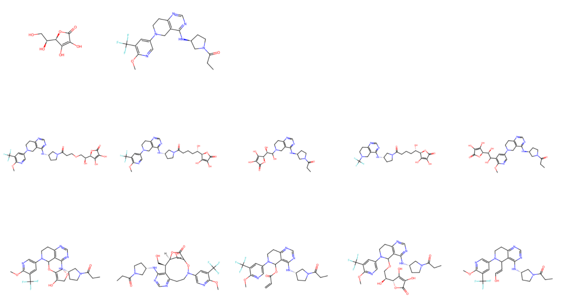

(d)

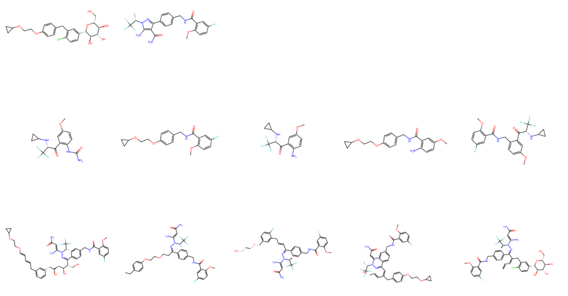

(e)

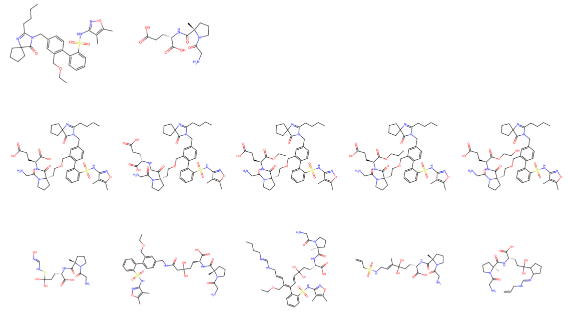

(f)

Figure 6. Using GPT-3.5-turbo to reproduce/mix molecules. Two original parent molecules on 1st row, followed by LLM created children, followed by conventional GA string splicing children for comparison

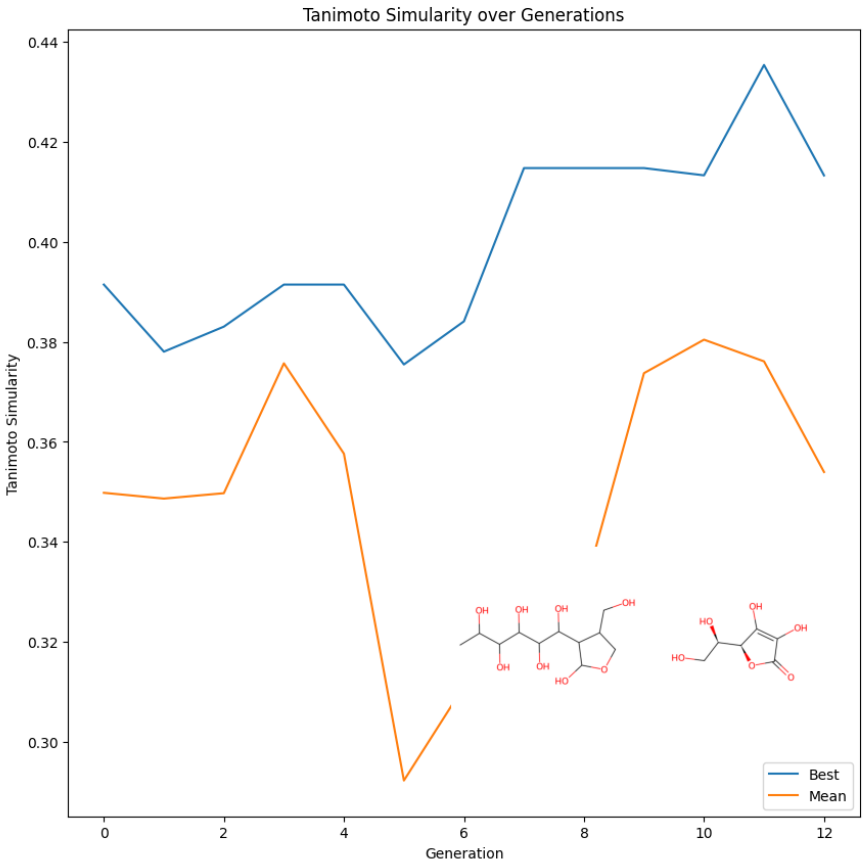

Figure 7. Tanimoto similarity to vitamin C as a function of GA generations. Conventional GA run for 30 generations and the best score (most similar to vitamin C) of each generation is given to the LLM as a LLM along with its associated Tanimoto similarity score to Vitamin C. LLM was then asked to create new molecules and improve the score for 12 generations. Multiple new best molecules were found using LLM as shown by the blue line.

II. Automation and novel interfaces

A. Using chain-of-thought and chemical tools to answer materials questions

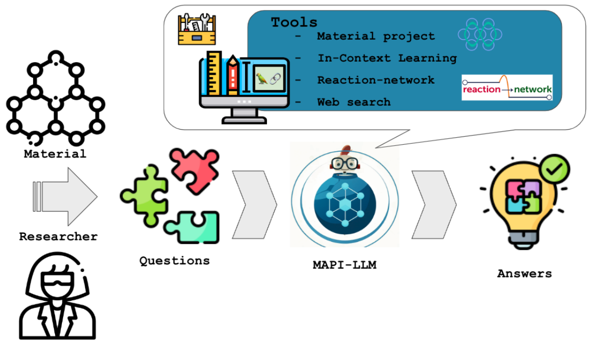

Figure 8. *Schematic overview of the MAPI-LLM workflow.* It uses LLMs to process the user's input and decide which available tools (e.g., Materials Project API, and Google Search) to use following an iterative chain-of-thought procedure. In this way, it can answer questions such as "Is the material AnByCz stable?".

LLMs have demonstrated remarkable success in various tasks [34–36]. Recently, LLMs have gained attention in chemistry, demonstrating exceptional ability to model chemical systems [37] and predicting tabular data [8, 22, 27]. Predicting the properties of materials is challenging since it requires computationally intensive techniques, such as density functional theory (DFT) [38–40]. Data-driven models offer a viable option to balance accuracy and computational time. Here we presented the MAPI-LLM, a multi-task package that employs LangChain [41] agents with access to multiple tools to address users' questions about materials.

It has been shown that providing chemistry-specific tools to an LLM allows the LLM to solve chemistry problems with significantly higher accuracy [42]. In a similar manner, we developed tools to iteratively query the Materials Project (MAPI) dataset [43] and utilize the reaction-network package [44], among others. MAPI-LLM can process user prompts in natural language using LLMs and follow a chain of thought (COT) [45] approach to determine the most suitable tools and inputs to answer the prompt. Due to MAPI-LLM's design, more tools can be added as needed, and tools can be combined (multiple tools can be used for a given prompt), opening the door for a large variety of applications. Figure 8 illustrates MAPI-LLM's capabilities. The code for the app is available in `https://github.com/maykcaldas/MAPI_LLM`, and a graphical user interface (GUI) is implemented in `https://huggingface.co/spaces/maykcaldas/MAPI_LLM`.

An important feature implemented into MAPI-LLM is a technique known as ICL [9], which allows the model to learn from the context within the prompt. For example, users can use MAPI-LLM's tool to query the MAPI dataset, first triggering the dataset search in the COT. However, if the desired material is not found in the dataset, MAPI-LLM still has access to other tools (such as ICL) to build context around the user prompt and adjust the COT actions to make a prediction. Another interesting tool is the ability to use the reaction-network package [44], which is a package for predicting inorganic reaction pathways. We showed the promising capabilities of MAPI-LLM by simply asking for reactions that use a given material as reactants or products. It can suggest such reactions for material synthesis or decomposition.

We built from the knowledge that LLMs are suitable for such tasks of interest in this application, for instance, classification and regression tasks [8]. Nevertheless, this application still needs a systematic validation of its predictions, such as the reinforcement learning from human feedback (RLHF) implementation in GPT-3.5 [46].

One sentence summaries

a. Problem/Task Answering complex materials science questions based on reliable data and tools.

b. Approach LLM-based agent in the ReAct framework that has access to tools such as the Materials Project API and uses ICL to answer questions for materials that are not in the materials project.

c. Results and Impact Coupling of tools allows answering questions that none of the tools or LLMs alone could solve by themselves, providing a very accessible interface to materials informatics tools.

d. Challenges and Future Work If a description of tools is incorporated in the prompt, this limits the number of tools that can be coupled. In addition, LLM agents still tend to not perform equally well on all prompts, and systematic investigation to better understand this and to increase the robustness is needed.

B. sMolTalk

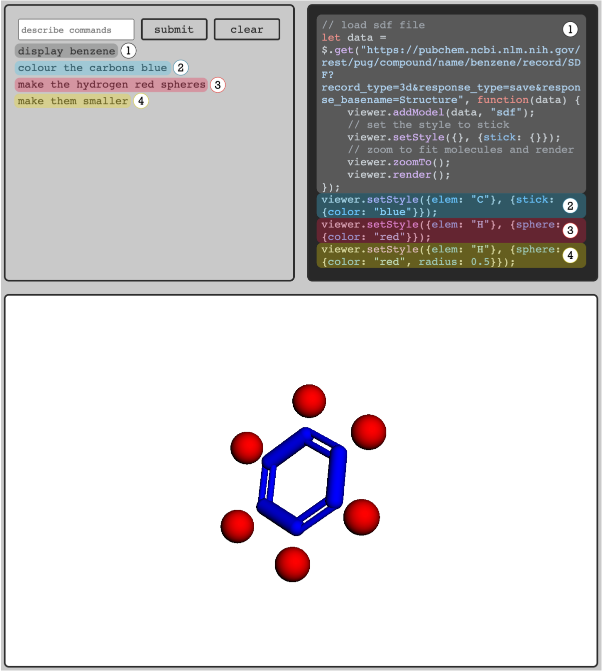

Figure 9. *The sMolTalk interface.* Based on few-shot prompting LLMs can create code for visualization tools such as `3dmol.js`.

Since the advent of 3D visualization methods, chemists have employed computers to display their molecules of interest to better understand their underlying structure and properties. Nevertheless, a lot of chemists are not equipped with the required coding skills to use and customize their visualizations. Depending on the package, and its associated documentation, chemists might end up spending hours to days learning the details of the specific visualization software.

We developed a natural language interface that generates code for `3dmol.js`, an open-source visualization JavaScript library [47], meaning the visualizations are run in a web browser (Figure 9). The user input is fed into ChatGPT API, using the GPT-3.5-turbo model. We use in-context learning (few-shot prompting), giving several examples of the user input with the expected JavaScript code that manipulates the `3dmol.js` viewer. Before the user submits further commands, we update the prompt with the current state of the viewer.

The current implementation might lead to a one-stop solution for visualizing and retrieving properties for molecules. This would accelerate chemists' workflow for querying information about molecules. Furthermore, if an LLM is able to control structural software, it might be possible to perform reasoning on the molecular structure itself. For instance, in drug discovery, one may ask what functional group of the ligand needs to be changed for binding affinity to the protein to increase. Another example might involve proteins, looking at what amino acid residues could be mutated to cysteines in order to create new disulfide bonds between chains. This would presumably require specific fine-tuning and equipping the LLM with more tools. The approach of generating code and structural reasoning might be similar but is most likely going to require a different set of tools that were specifically developed for protein structure manipulation (such as `PyMoL` [48], or `MolStar` [49]). Then, another set of highly accurate tools for binding affinity predictions or protein folding is also required. The major problem encountered is prompt leakage, where examples from in-context learning would leak into the actual LLM output. For the best evaluation, it is best to have as few and as different examples as possible. Moreover, although OpenAI's GPT models can sometimes correctly recall protein data bank (PDB) IDs of proteins or Chemical Abstract Services (CAS) numbers of compounds, it's not reliable, making tooling the models with API calls to PubChem, or the PDB, much more robust. We are currently developing an agent based on the ReAct approach [50] tooled with these APIs so that correct structures are always retrieved (i.e., to avoid the LLM needs to remember internally all such IDs). This framework would

then help us iteratively add tools to the agent, creating a chatbot one can query about any molecule of interest, including the structural reasoning task mentioned above. Lastly, we hypothesize we could improve the generation of `3dmol.js` code by using self-instruct fine-tuning. Using an external LLM with access to the documentation would create a dataset that could be used for fine-tuning. The same approach might be utilized for generating code for any other type of software, not just visualization packages. Therefore, such LLM could control molecular dynamics software, such as `LAMMPS` [51], or `GROMACS` [52].

One sentence summaries

a. Problem/Task Making bioinformatics tools, in particular the visualization software `3dmol.js` accessible to non-experts.

b. Approach Chat-interface by prompting a LLM to produce commands to `3dmol.js`, which are then passed to the software.

c. Results and Impact The LLM can, without consulting the documentation, generate code that often successfully performs the requested actions, demonstrating that LLM might help make tools more accessible by providing access to complex interfaces via natural language.

d. Challenges and Future Work The approach suffers from robustness issues, which might be overcome with conditioning on a knowledge base, more advanced decoding strategies, or error handling (e.g., giving the agent access to error messages and logs and prompting it to reflect).

C. `whinchat`: A Conversational ELN Interface

Laboratory data management systems such as ELNs and laboratory information systems (LIMS) can record rich data about experimental samples and the experiments that have been performed on them. Then, the user interfaces for these systems typically allow researchers to explore their samples with various pre-programmed visualizations. However, it is difficult for a traditional user interface to have enough flexibility to capture the richness and diversity of real, interconnected, experimental data. Here, our aim was to investigate whether an LLM could process digital laboratory data and serve as a flexible, conversational interface to a data management system. As a proof-of-concept for this approach, we introduced an automated data management assistant into the open-sourced "Datalab" software, which we have been developing to address needs in experimental materials chemistry (`https://github.com/the-grey-group/datalab`). Datalab can be used to capture data and metadata about materials chemistry samples, their synthesis constituents, and resulting devices. The system tracks each sample's provenance through the research lifecycle and makes this data available via a JavaScript object notation (JSON) API that can be explored programmatically or with a Vue.js-based web user interface (UI). During the hackathon, we embedded a prompted LLM into the UI and gave it access to raw responses from datalab's JSON API. We used the most recent publicly available models from OpenAI (gpt-3.5-turbo) via their Python API. We found that the LLM does a very good job at interpreting JSON and inferring the meaning of fields (including linked data) and can:

1. *Contextualize existing data:* Explain a set of related experiments from linked JSON responses, resolve acronyms/ short-hand used by experimentalists or create simple text summaries.

Contextualizing existing data

Assistant Hello! I am your virtual assistant and I am here to help you manage your experimental data. How can I assist you today?

User Can you provide a brief summary of this experiment?

Assistant Based on the provided JSON data, this experiment involves the synthesis and characterization of $NaCoO_2$ as a battery cathode material. The synthesis method used was solid-state reaction, and the starting materials used were sodium carbonate and cobalt (II,III) oxide. The synthesized $NaCoO_2$ was then used to make an electrode, which was characterized using X-ray diffraction. The electrode was then incorporated into a coin cell battery, which was cycled electrochemically and characterized using various techniques.

2. *Generate text and graphical* representations of the data: Aggregate data from different fields in the JSON response and convert to formats such as tables (potentially formatted for a particular journal), CSV, and even mermaid.js graph visualizations.

3. *Suggest future experiments:* The assistant readily identifies related materials to study, additives to try, additional characterization techniques, and synthesis protocols.

Suggest experiments

User: What NMR nuclei could I probe in this sample?

Assistant: Based on the information provided in the JSON file, the sample is $NaCoO_2$, which contains sodium, cobalt, and oxygen. Therefore, the NMR nuclei that could be probed in this sample are:

(a) Sodium-23 (23Na) NMR: This can provide information about the local environment and coordination of sodium ions in the $NaCoO_2$ lattice.

(b) Cobalt-59 (59Co) NMR: This can provide information about the local environment and coordination of cobalt ions in the $NaCoO_2$ lattice.

(c) Oxygen-17 (17O) NMR: This can provide information about the local environment and coordination of oxygen ions in the $NaCoO_2$ lattice.

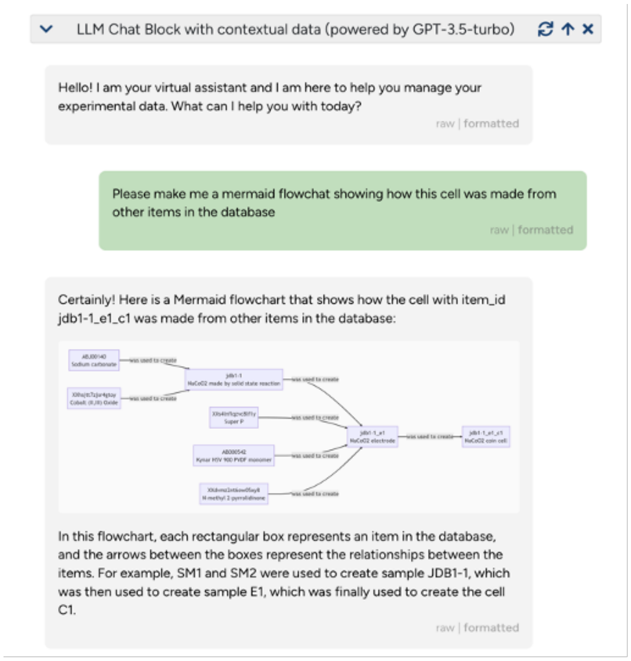

Figure 10. The chatbot can generate mermaid.js markdown code, which our interface automatically recognizes and translates into the visualization.

> It is worth noting that NMR spectroscopy is a powerful technique for probing the local environment of atoms in a material, but it requires a relatively large amount of sample and may not be sensitive to all types of nuclei. Therefore, it may be necessary to use other techniques in conjunction with NMR to fully characterize the sample.
>
> **User:** What techniques (that I haven't already used) should I use in conjunction with the NMR?
>
> **Assistant:** In addition to the techniques I mentioned earlier, you could use the following techniques in conjunction with NMR to fully characterize the $NaCoO_2$ sample:
>
> (a) X-ray photoelectron spectroscopy (XPS): This can provide information about the chemical composition and oxidation state of the $NaCoO_2$ sample, which can help to interpret the NMR spectra.
>
> (b) Transmission electron microscopy (TEM): This can provide information about the crystal structure, morphology, and defects of the $NaCoO_2$ sample, which can help to interpret the NMR spectra.
>
> (c) Electrochemical impedance spectroscopy (EIS): This can provide information about the electrical properties and performance of the $NaCoO_2$ sample as an electrode material, which can help to interpret the NMR spectra.
>
> (d) Thermogravimetric analysis (TGA): This can provide information about the thermal stability and decomposition behavior of the $NaCoO_2$ sample, which can help to interpret the NMR spectra.
>
> By using a combination of these techniques, you can gain a more complete understanding of the $NaCoO_2$ sample

The assistant performs quite well at certain tasks out-of-the-box, but we believe it should also be possible to further increase its general utility by fine-tuning the model on the domain knowledge present in a research

group's Wiki and papers. Furthermore, while we have only given the model data from a single project so far (up to 8 samples/starting materials, 1700 tokens of JSON), it would be of great interest to provide the model with a larger context across multiple projects to attempt to facilitate cross-fertilization of ideas. One notable challenge in this area is the limited context size of currently available LLM models (e.g., 4097 tokens for GPT-3.5-turbo). Therefore, future work will investigate larger models (e.g., GPT-4 with 30K token context), as well as approaches to give existing LLMs access to larger context (e.g., an embedding-based approach or allowing an LLM agent to query the OpenAPI directly as needed). At present, we note that the scientific usefulness of this assistant is highly task- and model-dependent; however, any additional interface that can lower the barrier to improving data capture and dissemination in the field should be investigated further and will be a future development target for Datalab.

One sentence summaries

a. Problem/Task Providing very flexible access to data in ELNs/LIMS.

b. Approach Prompting of a large language model with questions provided in a chat interface and context coming from the response of the API of an LLM.

c. Results and Impact The system can successfully provide a novel interface to the data and let user interact with it in a very flexible and personalized way, e.g, creating custom summaries or visuals for which the developers did not implement specific tools.

d. Challenges and Future Work Since the current approach relies on incorporating the response of the ELN/LIMS into the prompt, this limits how much context (i.e., how many experiments/samples) the system can be aware of. One potential remedy is to use retrieval-augmented generation, where the entries are embedded in a vector store and the agent will be able to query this database on put (parts of) the most relevant entries into the prompt.

D. BOLLaMa

The field of chemistry is continuously evolving towards sustainability, with the optimization of chemical reactions being a key component [53]. The selection of optimal conditions, such as temperature, reagents, catalysts, and other additives, is challenging and time-consuming due to the vast search space and high cost of experiments [54]. Expert chemists typically rely on previous knowledge and intuition, leading to weeks or even months of experimentation [55].

Bayesian optimization (BO) has recently been applied to chemistry optimization tasks, outperforming humans in optimization speed and quality of solutions [55]. However, mainstream access to these tools remains limited due to requirements for programming knowledge and the numerous parameters these tools offer. To address this issue, we developed BOLLaMa. This artificial intelligence (AI)-powered chatbot simplifies BO for chemical reactions with an easy-to-use natural language interface, which facilitates access to a broader audience.

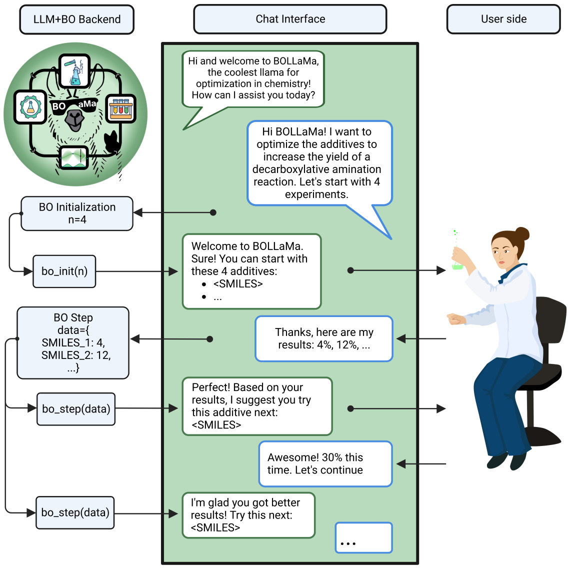

Figure 11. *Schematic overview of BOLLaMa.* A LLM can act as an interface to a BO algorithm. An experimental chemist can bootstrap an optimization and then, via a chat interface, update the state of the simulation to which the bot responds with the recommended next steps.

BOLLaMa combines LLMs with BO algorithms to assist chemical reaction optimization. The user-friendly interface allows even those with limited technical knowledge to engage with the tool. BOLLaMa's current implementation provides two main tools: the initialization function and the optimization step function [56], that are retrieved on LLM-demand as shown in Figure 11.

The primary contribution of this project is democratizing access to advanced BO techniques in chemistry, promoting widespread adoption of sustainable optimization tools, and impacting sustainability efforts within the community. This approach can be further enhanced to provide a more comprehensive assistant experience, such as with additional recommendations or safety warnings, and improve the explainability of the BO process to foster user trust and informed decision-making.

Key insights gained from this project include the critical role of accessibility in developing expert tools and the potential of LLMs in chemistry through various agent architectures [50]. In addition, the initial BO tool adapted for BOLLaMa was designed for closed-loop automated laboratories, emphasizing the need for accessible tools catering to diverse user backgrounds.

One sentence summaries

a. Problem/Task Giving scientists without coding and machine learning expertise access to Bayesian optimization.

b. Approach LLM as a chat-interface for a Python package for Bayesian optimization by using ReAct-like approach in which the LLM has access to text-description of relevant functions (such as initialization and stepping of the BO run).

c. Results and Impact The chat interface can successfully initialize a BO run and then convert observations reported in natural language into calls to the stepping function of the BO tool.

d. Challenges and Future Work As most LLM agents, the tools suffers from robustness issues and the correct functioning cannot be guaranteed for all possible prompts.

III. Knowledge Extraction

A. InsightGraph

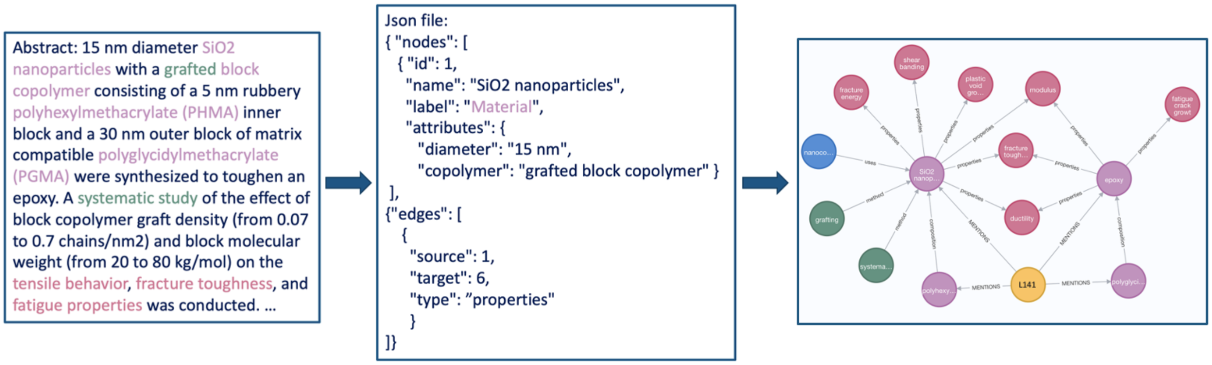

Figure 12. *The Insight Graph interface.* A suitably prompted LLM can create knowledge graph representations of scientific text that can be visualized using tools such as neo4j's visualization tools. [57]

The traditional method of performing a literature review involves months of reading relevant articles to find crucial information on material properties, structure, reaction pathways, and applications. Knowledge graphs are sources of structured information that enable data visualization, data discovery, insights, and downstream machine-learning tasks. Knowledge graphs extracted from published scientific literature covering broad materials science domains [58] as well as more-focused domains such as polymer nanocomposites [59] empower material scientists to discover new concepts and accelerate research. Until recently, capturing complex and hierarchical relationships for a knowledge graph within the materials science literature was a time-consuming effort, often spanning multi-disciplinary collaborations and many Ph.D. years. By leveraging zero to few-shot training and pre-trained LLMs, it is now possible to rapidly extract complex scientific entities with minimal technical expertise [58, 60, 61]. We envision that knowledge graphs built by LLMs based on scientific publications can offer a concise and visual means to launch a literature review.

To demonstrate a proof of concept of a zero-shot entity and relationship extraction, we identified 200 abstracts on polymer-nanocomposite materials for which detailed structured information was already available [62]. Each abstract was fed as a prompt to GPT-3.5-turbo, a language model powering the popular ChatGPT web application by OpenAI. The instructions in our prompt consisted of an example JSON containing high-level schema and information on possible entities and pairwise relationships. The nodes and relationships in the output JSON response were then stored in a neo4j graph database using Cypher, a graph query language (Figure 12). [57] The zero-shot capabilities of the model allowed the specification of an arbitrary entity and relationship types depending upon the information contained in the text. Given that this required a change in the neo4j pipeline every time the prompt changed, we found it necessary to constrain the JSON schema to a standard format.

While large language models on their own are prone to hallucinations, leveraging them with guidance to create structured databases empowers chemists/materials scientists with no expertise in natural language processing to search and build on existing knowledge leading to new insights. The speed at which LLMs can create structured graphs dramatically exceeds the years required for humans to manually curate data into existing knowledge graphs. Access to structured databases will accelerate the pace of data-driven material science research, synthesizing details embedded in dispersed scientific publications. Additionally, other scientific fields could benefit from a similar use of LLMs to extract entities and relationships to build knowledge graphs.

Owing to the non-deterministic nature of LLMs, we found that the output response would vary even when the same prompt was provided. An instruction constraining the JSON schema minimized the variability. A systematic study comparing different foundation models, prompt techniques (zero-shot, one-shot, few-shot), prompt chaining, and the role of fine-tuning is needed to evaluate the precision and recall of extracted entities

and relationships. Notably, pairwise links between the nodes are not often enough to model the complex nature of materials requiring improvement in the input schema.

One sentence summaries

a. Problem/Task Extraction of entities and their relationships from text.

b. Approach Prompting of GPT-3.5-turbo prompted with abstract and example JSON and the task to extract entities and their relationships in a structure as provided in the example.

c. Results and Impact The approach can successfully create meaningful JSON data structures with extracted entities and their relationships for hundreds of abstracts.

d. Challenges and Future Work The non-deterministic behavior of LLMs can lead to variability and fragile behavior. To better understand this as well as the performance of this approach, more systematic benchmarking is needed.

B. Extracting Structured Data from Free-form Organic Synthesis Text

A suspension of compound 63 (0.22 g, 0.93 mmol) and 64 (0.33 g, 0.92 mmol) in THF/triethylamine (11 mL, 10/1) was stirred at room temperature for 48 h. After this time, a clear solution was formed. The solvent was removed under reduced pressure and the residue was purified by flash chromatography (silica gel, 10:1:0.1 chloroform/methanol/concentrated ammonium hydroxide) to provide the guanidine 65 (0.3 g, 60%) as a yellow solid. 1H NMR (300 MHz, DMSO-d6) δ 1.42 (s, 9H), 1.55 (m, 4H), 2.19 (s, 6H), 2.58 (m, 4H), 3.99 (m, 2H), 6.83 (d, 2H), 7.12 (d, 2H), 7.40 (br s, 2H), 9.02 (m, 2H).


```
{
  "m1_m2_m5": {
    "components": [
      {
        "identifiers": [{"type": "NAME", "value": "compound 63"}],
        "amount": {"mass": {"value": 0.22, "units": "GRAM"}},
        "reactionRole": "REACTANT"
      },
      {
        "identifiers": [{"type": "NAME", "value": "compound 64"}],
        "amount": {"mass": {"value": 0.33, "units": "GRAM"}},
        "reactionRole": "REACTANT"
      },
      {
        "identifiers": [{"type": "NAME", "value": "THF"}],
        "amount": {"volume": {"value": 11, "units": "MILLILITER"}},
        "reactionRole": "SOLVENT"
      }
    ]
  },
  "m3": {
    "components": [
      {
        "identifiers": [{"type": "NAME", "value": "triethylamine"}],
        "amount": {"volume": {"value": 11, "units": "MILLILITER"}},
        "reactionRole": "SOLVENT"
      }
    ]
  }
}
```


Figure 13. *The Organic Synthesis Parser interface.* The top part shows text describing an organic reaction (`https://open-reaction-database.org/client/id/ord-1f99b308e17340cb8e0e3080c270fd08`), which the finetuned LLM converts into structured JSON (bottom). A demo application can be found at `https://qai222.github.io/LLM_organic_synthesis/`.

a. Problem As data-driven approaches and machine learning (ML) techniques gain traction in the field of organic chemistry and its various subfields, it is becoming clear that, as most data in chemistry is represented by unstructured text, the predictive power of these approaches is limited by the lack of structured, well-curated data. Due to the large corpus of organic chemistry literature, manual conversion from unstructured text to structured data is unrealistic, making software tools for this task necessary to improve or enable downstream applications, such as reaction prediction and condition recommendation.

b. Solution In this project, we leverage the power of fine-tuned LLMs to extract reactant information from organic synthesis text to structured data. 350 reaction entries were randomly selected from the Open Reaction Database (ORD) [63]. The field of `reaction.notes.procedure_details` is used as the input (prompt), and the field of `reaction.inputs` is used as the output (completion). 300 of these prompt-completion pairs were used to fine-tune a GPT-3 (OpenAI Davinci) model using the OpenAI command line interface (version 0.27.2), and the rest were used for evaluation. In addition to this, we also explored fine-tuning the Alpaca-LoRA model [16, 64, 65] for this task. All data and scripts used in this project are available in the GitHub repository.

c. Results and Discussion Surprisingly, the pre-trained language model (OpenAI Davinci), fine-tuned with only 300 prompt-completion pairs, is capable of generating valid JSON complying with the ORD data model. For the 50 prompt-completion pairs in evaluation, 93 % of the components in reaction inputs were correctly extracted from the free text reaction description by the GPT-3 based model. The model also associates existing properties, such as volume or mass used in the reaction, to these components. In addition to recognizing in-text chemical entities (such as molecule names), as shown in Figure 13, tokens referencing external chemical entities (compound numbers) can also be captured by the model. On the other hand, while completing the prompts with extracted chemical information, the fine-tuned Alpaca-LoRA model was unable to properly construct a valid JSON complying with the ORD data model.

Despite these encouraging preliminary results, there are still challenges to a robust synthesis text parser. One of them is the ambiguous and often artificial boundary between descriptions of reactions and workups, which leads to misplaced chemical entities in the structured data, e.g., a solvent used in the extraction of products is instead labeled as a reaction solvent. The aforementioned external reference problem, where a compound number in the procedure is only explicitly identified in an earlier section of the manuscript, can only be solved by prompting the LLM with multiple paragraphs or even the entire document, adding more irrelevant tokens to the prompt. It is also important to prevent the LLM from "auto-completing" extracted named entities with information outside the prompt, e.g., the chemical is extracted as "sodium chloride" in the completion while it is only specified as "chloride" in the prompt.

One sentence summaries

d. Problem/Task Extraction of structured reaction condition and procedure data from text.

e. Approach Fine-tuning of LLMs on hundreds of prompt (unstructured text)- completion (extracted structured data) pairs.

f. Results and Impact OpenAI's davinci model can extract the relevant data with a success rate of 93 %.

g. Challenges and Future Work Parameter efficient fine-tuning could not match the performance of OpenAI's models. In addition, there are instances in which the LLM goes beyond the specified tasks (e.g., modifies/"autocompletes") extracted entries, which can lead to fragile systems.

C. TableToJson: Extracting structured information from tables in scientific papers

Much of the scientific information published in research articles is presented in an unstructured format, primarily as free text, making it a difficult input for computational processing. However, relevant information in scientific literature is not only found in text form. Tables are commonly employed in scientific articles, e.g., to collect precursors and raw materials' characteristics, synthesis conditions, synthesized materials' properties, or chemical process results. Converting this information into a structured data format is usually a manual time-consuming and tedious task. Neural-network-based table extraction methods and optical character recognition (OCR) [66], which can convert typed, handwritten, or printed documents into machine-encoded text, can be used to extract information from tables in PDF files. However, it is often not straightforward to extract the data in the desired structured format. Nonetheless, structured data is essential for creating databases that aggregate research results, and enable data integration, comparison, and analysis.

In this context, JSON is a widely adopted structured data format due to its simplicity, flexibility and compatibility with different programming languages and systems. However, obtaining structured data following a specific JSON schema with models can be challenging. The generated JSON needs to be syntactically correct and conform to a schema that defines the JSON's structure. Models typically do not provide structured output that perfectly matches the desired JSON schema. Some manual post-processing or data transformation is often necessary to map the extracted information to the appropriate schema fields.

In this work, we have studied two approaches to generate structured JSON from data contained in tables of scientific papers focused on different research topics within the field of chemistry [67–73]. The Python `json` module was used to parse JSON data and validate the outputs.

As a first approach, the OpenAI `text-davinci-003` model was used to generate structured JSON from data in tables. The input to the LLM is the HyperText Markup Language (HTML) code of the table, obtained directly from the digital object identifier (DOI) of the article using the Python `selenium` library, while the output of the model is the data extracted in JSON form (Figure 14). The OpenAI `text-curie-001` model, although not tested in this work, can also be utilized if the number of input tokens, considering both the HTML text of the table and the schema, meets the requirements of this model (máximum 2049 input tokens, compared to 4097 for `text-davinci-003`).

The use of the OpenAI model to generate structured JSON was compared with a second approach, i.e., the use of `jsonformer` (`https://github.com/1rgs/jsonformer`), which implements a data processing pipeline that combines the model generation with appropriate data transformation. This method introduces an efficient way to generate structured JSON using LLMs by generating only the content tokens and filling in the fixed tokens. This avoids generating a complete JSON string and parsing it. This approach ensures that the produced JSON is always syntactically correct and aligns with the specified schema. [74]

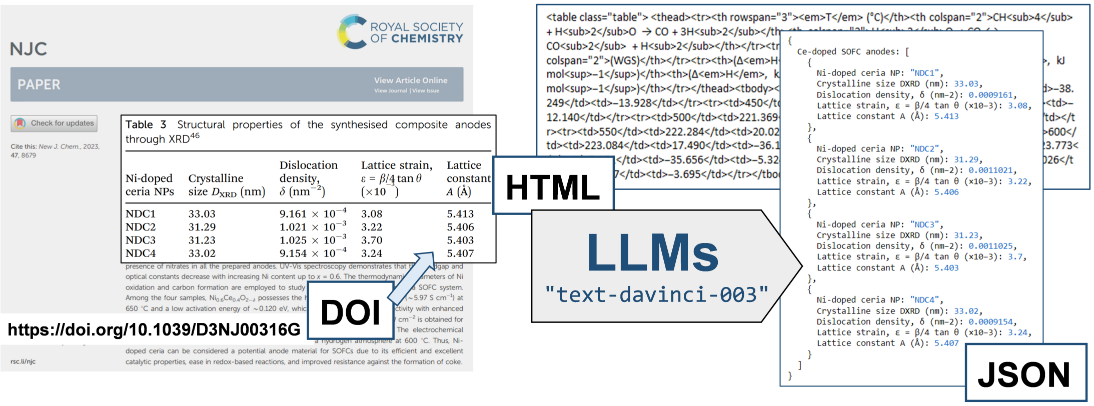

Figure 14. *TableToJson.* Extraction of structured information from scientific data in tables using LLMs. The input to the LLM model is the HTML code of a table contained in a scientific paper. The output of the LLM model is data structured in JSON form. Results can be visualized in this demo app: `https://vgvinter-tabletojson-app-kt5aiv.streamlit.app/`.

In our first approach, we directly asked the OpenAI `text-davinci-003` model to generate a JSON object according to a desired JSON schema provided in the model prompt. The table content was also included in

the prompt as HTML code. The accuracy in the prediction, calculated as the percentage of schema values generated correctly, is shown in Figure 15. In all examples, the OpenAI model was queried with a simple prompt, and it correctly extracted all the data in the table and inserted every value into the corresponding position in the schema, with 100 % accuracy, providing as output a JSON object. This model also correctly generated both string and number values according to the type assigned in the schema. However, in two of the examples, the OpenAI model did not generate the JSON object name specified in the schema when the corresponding name was not found in the table, generating only the list of components. This was solved by modifying the object name in the schema to a term that more closely aligned with the content of the table. It appears that when the model could not establish a clear relationship between the provided name and the table content, it disregards that part of the schema during generation. These results indicate that the OpenAI `text-davinci-003` model is able to convert scientific data from tables of research papers to a structured format following the approach used in this work, where the desired JSON schema was included in the model prompt. Nevertheless, the model retains a certain degree of freedom to modify the requested scheme if it considers that something may be wrong.

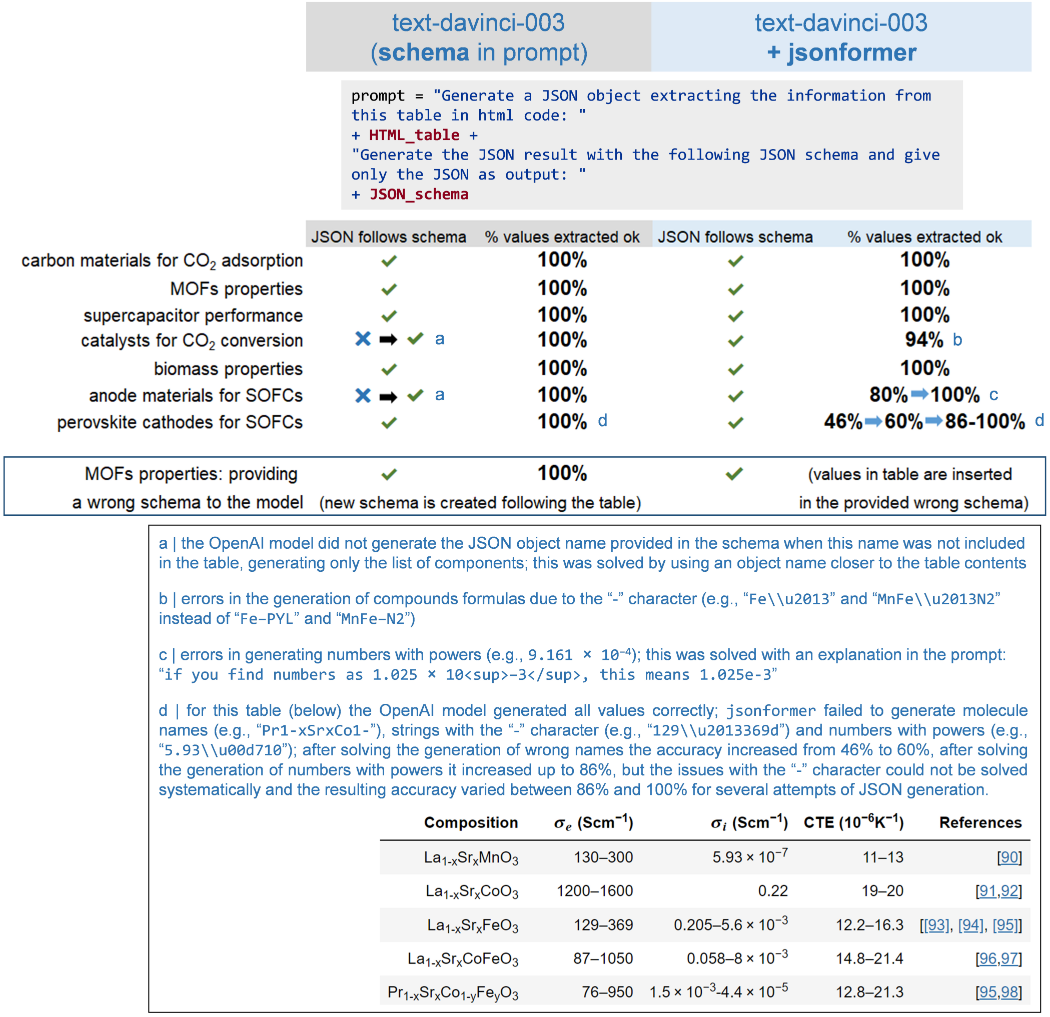

Figure 15. *TableToJson.* Results of the structured JSON generation of tables contained in scientific articles. Two approaches are compared: (i) the use of an OpenAI model prompted with the desired JSON schema, and (ii) the use of an OpenAI model together with `jsonformer`.

The second approach used to generate structured information was a version of the `jsonformer` approach adapted for use with OpenAI LLMs (`https://github.com/martinezpl/jsonformer/tree/add-openai`), with the implementation of the inclusion of the table text as an input parameter to the `jsonformer` function.

Detection of strings indicating null values was also added when the schema type is number, as "nan", "NaN", "NA", and "NAN" entries are common in research data tables. The OpenAI `text-davinci-003` model was used. In this case, the model was prompted with the desired JSON schema and the HTML code of the studied table. `Jsonformer` reads the keys from the JSON schema and only delegates the generation of the value tokens to the language model, ensuring that a valid JSON is generated by the LLM model.

For this approach, the accuracy in the prediction is also shown in Figure 15. The use of the OpenAI `text-davinci-003` model together with `jsonformer` generated valid JSON objects with 100% accuracy for most of the tables evaluated using a simple prompt. Figure 16 shows the results of one of the examples studied, where using a simple descriptive prompt denoting the type of input text, this approach correctly generated structured data JSON from a table with a complex header. However, it was detected that when the values to be generated contain special characters or specific texts, a more detailed prompt with some simple examples, but without finetuning, can be necessary to provide good results, as shown in Figure 17 for a special numeric notation that included power numbers.

					Ultimate analysis				Proximate analysis	HHV	H/O	He density
Sample					(wt%, db)	(wt%)			(wt%, db)	(MJ/kg, db)	ratio	(g/cm³)[b]
	C	N	H	S	O[a]	MC	Ash	VM	FC[a]	Empty Cell	Empty Cell	Empty Cell
AS	49.44	0.31	5.85	0.05	42.90	6.5	1.45	78.9	19.6	19.565	2.16	1.252
CHE	50.22	0.34	5.55	0.01	43.41	8.4	0.47	81.2	18.3	19.109	2.03	1.268
CHET	51.30	0.40	5.40	0.02	42.59	8.2	0.29	80.0	19.7	19.588	2.01	1.275
CS	47.96	2.74	5.93	0.21	35.26	6.7	7.90	70.4	21.7	19.067	2.67	1.156
GP	45.50	1.82	5.05	0.17	34.73	11.6	12.73	67.6	19.7	18.682	2.31	1.210
OS	51.21	0.29	6.01	0.03	41.88	4.3	0.58	81.5	17.9	20.511	2.28	1.241
PCL	52.89	0.44	6.06	0.03	39.46	10.1	1.12	76.5	22.4	20.976	2.44	1.237

```
prompt="Generate an object with the
following schema extracting the
information from the provided table
in html code:"
```


```
{
  Biomass type: [
    {
      Sample: "AS",
      Ultimate Analysis (wt%, db): {
        C: 49.44,
        N: 0.31,
        H: 5.85,
        S: 0.05,
        O: 42.9
      },
      Proximate Analysis (wt%, db): {
        MC (wt%): 6.5,
        Ash: 1.45,
        VM: 78.9,
        FC: 19.6
      },
      HHV (MJ/kg, db): 19.565,
      H/O: 2.16,
      He density (g/cm3): 1.252
    },
    {
      Sample: "CHE",
      Ultimate Analysis (wt%, db): {
        C: 50.22,
        N: 0.34,
        H: 5.55,
        S: 0.01,
        O: 43.41
      },
      Proximate Analysis (wt%, db): {
        MC (wt%): 8.4,
        Ash: 0.47,
        VM: 81.2,
        FC: 18.3
      },
      HHV (MJ/kg, db): 19.109,
      H/O: 2.03,
      He density (g/cm3): 1.268
    },
    {
      Sample: "CHET",
      Ultimate Analysis (wt%, db): {
        C: 51.3,
        N: 0.4,
        H: 5.4,
        S: 0.02,
        O: 42.59
      },
      Proximate Analysis (wt%, db): {
        MC (wt%): 8.2,
        Ash: 0.29,
        VM: 80.0,
        FC: 19.7
      },
      HHV (MJ/kg, db): 19.588,
      H/O: 2.01,
      He density (g/cm3): 1.275
    },
    {
      Sample: "CS",
      Ultimate Analysis (wt%, db): {
        C: 47.96,
        N: 2.74,
        H: 5.93,
        S: 0.21,
        O: 35.26
      },
      Proximate Analysis (wt%, db): {
        MC (wt%): 6.7,
        Ash: 7.9,
        VM: 70.4,
        FC: 21.7
      },
      HHV (MJ/kg, db): 19.067,
      H/O: 2.67,
      He density (g/cm3): 1.156
    },
```


Figure 16. *TableToJson.* Structured JSON generation of tables contained in scientific articles using a prompt with a simple description of the type of input text. One example is shown for a table that contains data on properties of biomass materials [71].

As shown in Figure 15, in one of these examples, an accuracy of 94 % was obtained from a table containing a few catalyst names that included the "-" character, and those values were erroneously generated. In another example, an accuracy of 80 % was initially obtained due to errors in the generation of numbers with powers (e.g., 9.161×10^4), which could be solved by adding an explanation in the prompt: `"if you find numbers as 1.025 × 10<sup>3</sup>, this means 1.025e-3"`, increasing the accuracy to 100 %.

Next, a table with more complex content (long molecule names, hyphens, power numbers, subscripts, and superscripts…) was selected (Figure 15), resulting in an accuracy of 46% in the JSON generation, meaning that only 46% of the schema values were correctly generated. The erroneous generation of long formula or molecule names with a mixture of letters and numbers as subscripts could be solved by increasing the value of the `max_string_token_length` argument of the `jsonformer` function to get a longer response where the end of the string can be detected more easily, which increased the accuracy to 60 %. `Jsonformer` also showed some issues in this example in generating power numbers, which are represented as `10<sup>—n</sup>` in the input HTML text. As mentioned above, this was solved by adding a specific explanation in the prompt, increasing the accuracy to 86%. A specific explanation was also included in the prompt to address the issues related to the presence of hyphens in the text. Still, this problem could not be solved systematically, and the resulting accuracy varied between 86 % and 100 % for several JSON generation attempts. In this particular case, the generated value provided by the model included Unicode text instead of the "-" character (and usually several "\" characters). An instruction to `"decode Unicode characters in your response"`

was then included in the prompt. Although this solution sometimes yielded satisfactory results, it did not systematically guarantee correct output. These results indicate that the OpenAI model combined with `jsonformer` can provide wrong outputs when the values to be generated contain some special characters, such as the "-" character in this example. This issue requires further investigation to be improved.

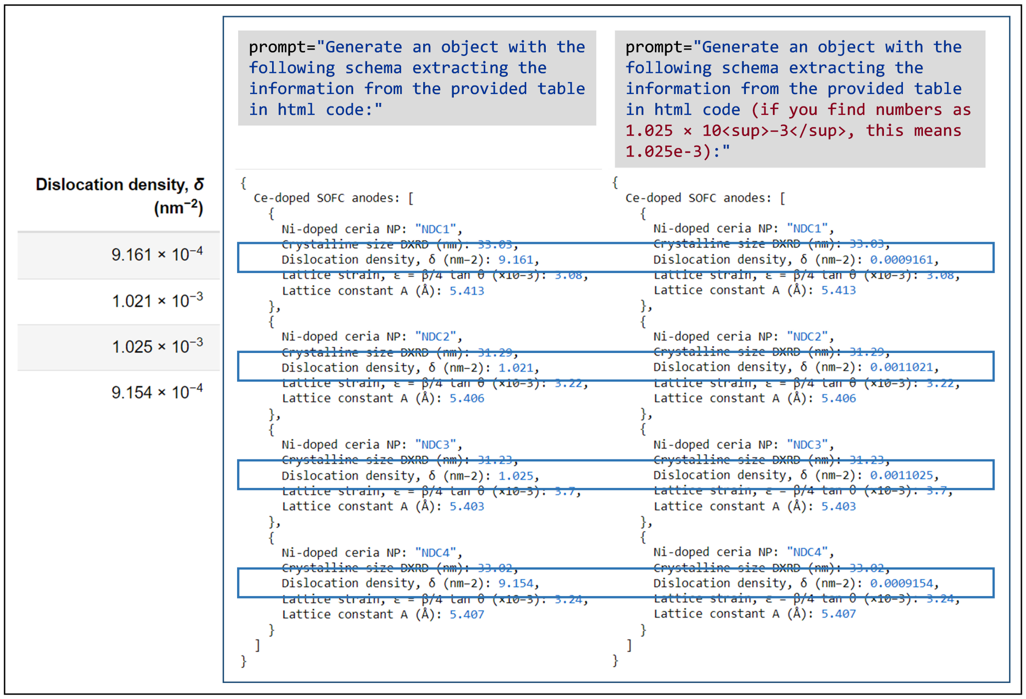

Figure 17. *TableToJson.* Structured JSON generation of a table contained in a scientific article using a standard prompt and a prompt with a few simple examples of the special numeric notation found in some of the cells of the input table [72].

Lastly, for one of the examples, a test was performed by providing a wrong schema to the model (Figure 15). In this case, as expected, `jsonformer` inserted the values contained in the table into the given wrong schema in a more or less ordered fashion, generating an invalid output. However, the OpenAI model created a new schema according to the table structure and headers, providing a valid result, and confirming its freedom to decide what may be wrong with the user's query. An example of these results is shown in Figure 18.

The two approaches used in this work showed a good performance in the generation of JSON format when the data contained in the table are regular strings or numbers, with an accuracy of 100 % in most of the examples. The results of this work show that, although the OpenAI `text-davinci-003` is able to easily extract structured information from tables and give a valid JSON output, this approach cannot guarantee that the outputs will always follow a specific schema. On the other hand, although `jsonformer` may present problems when special characters need to be generated, some of these issues have been solved with careful prompting, and others could probably be solved with further research. It can be concluded that `jsonformer` can be a powerful tool for the generation of structured data from unstructured information in most tables, ensuring the generation of valid JSON syntax as the output of LLMs that always complies with the provided schema. The use of `jsonformer` could facilitate and promote the creation of databases and datasets for numerous topics within the field of chemistry, especially in experimental domains, where the availability of structured data is very scarce.

One sentence summaries

a. Problem/Task Extracting structured data in a JSON-schema-compliant form from HTML tables.

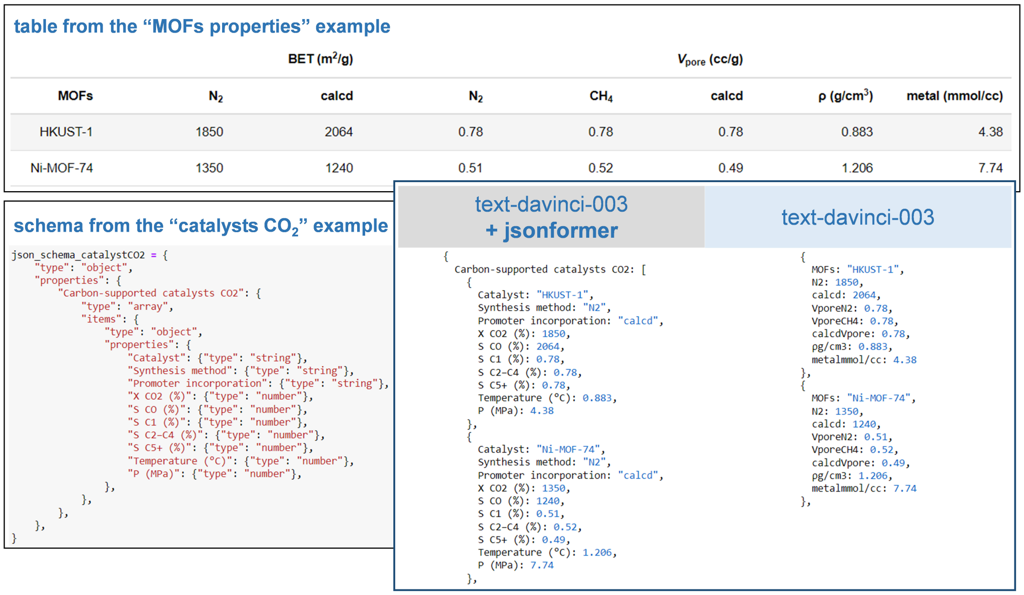

Figure 18. *TableToJson.* Results of the structured JSON generation of a table after providing the model with a wrong schema. The output generated using the OpenAI model together with `jsonformer` is shown on the left (values in the table are inserted in the provided wrong schema), while the output generated using directly the OpenAI model is shown on the right (a new schema is created following the table content).

b. Approach Two approaches were compared: Direct prompting of OpenAI's `text-davinci-003` model with the input table and the JSON schema, as well as the `Jsonformer` approach, which only samples from a subset of tokens in field-wise generation steps.

c. Results and Impact Both approaches can extract data in schema-compliant from tables with high success rates. Due to hard-coded decoding rules, `Jsonformer` failed in some cases.

d. Challenges and Future Work While the `Jsonformer` approach can guarantee valid syntax, it can fail in cases that were not considered in the development of the decoding rules. Hence, future work is needed for increasing the general applicability of constrained decoding strategies.

D. AbstractToTitle & TitleToAbstract: text summarization and text generation

1. *Problem*

Text summarization and text generation are some of most the common tasks in natural language processing (NLP). Often it is tricky to obtain well-defined and curated datasets for these tasks. Also, evaluating the performance of an NLP model is challenging because there is no unique way to summarize and generate text. Luckily, there are many publicly available manuscripts for chemistry and materials science in open access platforms such as arXiv and PubChem. These datasets can be used along with LLMs to solve problems such as: 1) given title of the article; generate an abstract, 2) given an abstract; generate a title, which should be similar to the one available in the literature. Such models can greatly help in technical writing, especially with a human expert in the loop.

2. *Solution*

The above challenging tasks require an organized infrastructure for curated data and tools. JARVIS-ChemNLP [75] is an integrated library for materials chemistry text data for NLP tasks such as text classification, token classification, abstractive summarization, text generation, and integrating with existing DFT databases. ChemNLP uses transformer models to generate titles from abstracts and vice versa for arXiv condensed matter category articles.

There is no unique approach for both tasks. Nevertheless, highly qualified scientists and engineers generate such abstracts and titles and can act as a valuable benchmark to evaluate model performance. Moreover, developing metrics for text generation is a difficult task. Here, for the sake of simplicity, we choose the Recall-Oriented Understudy for Gisting Evaluation (ROUGE) metrics for evaluating the model performance.

ChemNLP uses models such as a pre-trained Text-to-Text Transfer Transformer (T5) developed by Google for text summarization. The pre-trained model is further fine-tuned to produce summaries of abstracts and analyze the performance by analyzing its closeness to the title of the given paper.

For the text generation, ChemNLP fine-tunes LLMs such as the pre-trained GPT-2-medium model from the HuggingFace library.

For these tasks, the community can contribute their models on a benchmarking platform called the JARVIS-Leaderboard (`https://pages.nist.gov/jarvis_leaderboard/`). Specifically, the TextGen task is available at: `https://pages.nist.gov/jarvis_leaderboard/AI/TextSummary/arxiv_summary_text/` and TextSummary task is available at `https://pages.nist.gov/jarvis_leaderboard/AI/TextGen/arxiv_gen_text/`

3. *Example*

Title to Abstract example for `cond-mat/0105402`. [76]

Question

MgB2 under pressure: phonon calculations, Raman spectroscopy, and optical reflectance can be described as

Fine-tuned GPT-2-medium model output

The amplitude of phonon vibrations in a material exhibiting an unconventional lattice structure has been measured in the low pressure region of the granular MgB2 phase. The peak in the high pressure region is the first measurement of phonon amplitude ever presented in a granular superconducting material, and it indicates a potential source of superconductivity. Superconducting Transition from Fluctuations to Brillouin T-Wings in CeFo Josephson Junctions can be described as We report electronic and numerical calculations for solving the electron-wave (E-W) conduction spectra of a quasi-two-dimensional superconducting monolith CeFo Josephson junctions: The amplitude of

phonon vibrations in the low pressure region (KPSM), and in the high pressure region of the superconducting density wave (SDW) densities, are used to understand the superconductivity.

Actual abstract

The effect of pressure on optical phonon frequencies of MgB_2 has been calculated using the frozen-phonon approach based on a pseudopotential method. Gruneisen parameters of the harmonic mode frequencies are reported for the high-frequency zone-center E_{2g} and B_{1g} and the zone-boundary E_{2u} and B_{2u} modes at A. Anharmonic effects of phonon frequencies and the implications of the calculated phonon frequency shifts for the pressure dependence of the superconducting transition temperature of MgB_2 are discussed. Also reported are Raman and optical reflectance spectra of MgB_2 measured at high pressures. The experimental observations in combination with calculated results indicate that broad spectral features we observed in the Raman spectra at frequencies between 500 and 900 cm^{-1} cannot be attributed to first-order scattering by zone-center modes, but originate in part from a chemical species other than MgB_2 at the sample surface and in part from a maximum in the MgB_2 phonon density of states. Low-temperature Raman spectra taken at ambient pressure showed increased scattering intensity in the region below 300 cm^{-1}.

Interestingly, the generated abstract contains grammatically and syntactically incorrect sentences. We suspect that this is due to our use of a small, outdated, base model. However, more systematic analysis will need to be performed in future work.

One sentence summaries

a. Problem/Task Text summarization and generation, in specific, a summary of an abstract into a title and generation of an abstract conditioned on a title.

b. Approach Fine-tuning of transformer models such as T-5 and GPT-2 on data from arXiv.

c. Results and Impact Initial exploration indicates that transformer models might be suitable for this task.

d. Challenges and Future Work More systematic analysis, including rating of the generated titles and abstracts by domain experts is required to identify the limitations of this approach.

IV. Education

A. i-Digest

a. Problem Over the last few years, especially during the Covid period, most of us had to switch to the online mode of working in our day-to-day jobs. And even today, the online mode of working has, to some extent, stayed on as it turned out to be convenient for both employers and employees. One clear example can be found in the field of education, where the use of video lectures became the norm for teaching students in universities and schools. Likewise, podcasts and three-minute thesis videos, which communicate important scientific information to society at large, have grown tremendously [77, 78]. This has led to a situation where, at present, we have an enormous amount of important scientific information stored in the form of videos and audio all over the internet. A current challenge is to summarize and make use of this knowledge efficiently. Some efforts in this direction have been made by using AI Youtube summarizers and QnA Bots [79]. We would like to build upon such efforts and create a tool for the field of education.

b. Solution We present a tool that self-guides students and other users toward a better understanding of the content of a video lecture or a podcast. In order to accomplish this, we used publicly available LLMs like Open AI's Whisper [80] and GPT-3.5-turbo model. All the user needs to do is provide a link to the lecture video or audio file. After only a short time, the overview page shows some technical keywords on which the video is based, a short but comprehensive summary, and some questions for the user to assess his or her understanding of the concepts discussed in the video/audio (Figure 19). Additionally, for chemistry enthusiasts, if some chemical elements/molecules are discussed in the content, we link them to online databases. At the backend, we first convert the video to audio using Pytube (In the case of a podcast, this step is not needed). Then we use the Whisper model to transcribe the audio to text. Next, we make use of the OpenAI GPT-3.5-turbo model to obtain a short summary and a set of questions based on the text. Finally, we extract the name of chemical elements/molecules and list the PubChem database entry for that element/molecule on the overview page. [81–83] The web interface was made using the open-source app framework Streamlit [84].

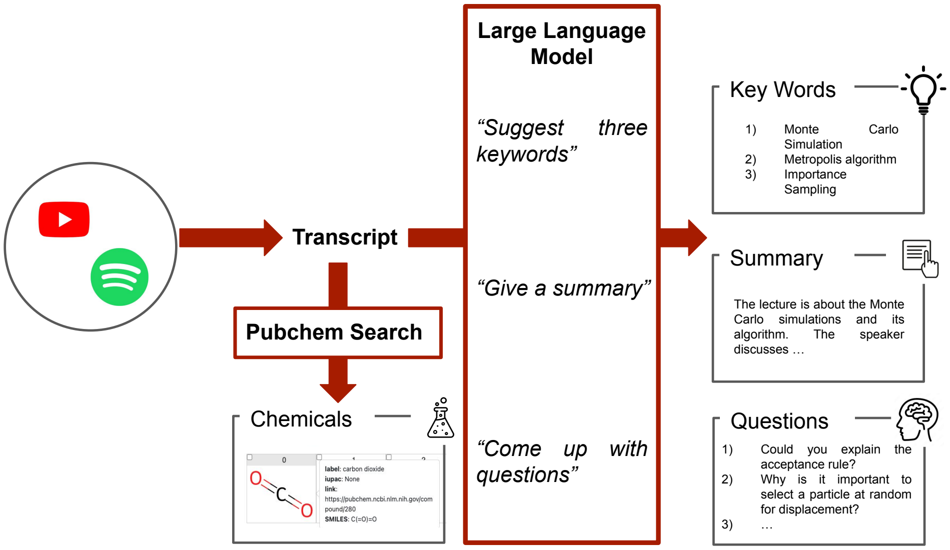

Figure 19. A schematic of the i-digest interface. On providing a link to an online video or audio, i-digest generates some technical keywords, a short but comprehensive summary, and a list of questions based on the content in the video/audio. Additionally, chemicals discussed in the content are linked to online databases such as PubChem.

c. Impact We strongly believe that extracting important scientific information in terms of short lecture notes and questions would help to push forward the field of education towards creating and using resources more efficiently. Moreover, by providing additional links to resources, e.g., databases, journals, and books,

we provide an opportunity for the user to go beyond the content of the lecture and spark interest in a more detailed understanding of the topic. Specifically, this would help researchers/teachers/professors to create new course content or to update/modify already available content. In general, our tool covers a broad range of users, from the youngest learner to the chemistry novice who wants to kickstart his research, all the way to professors, course creators, and lifetime learners.

d. Lessons learned Working together with colleagues can be fun and enriching and often help to solve big problems. This hackathon taught us that even in one day, coming together can help achieve something significant.

One sentence summaries

e. Problem/Task Provide students with automatically generated active learning tasks for lecture recordings.

f. Approach Transcription of videos using OpenAI's Whisper model, prompting of OpenAI's GPT-3.5-turbo model to produce a short summary and questions based on the transcript, as well as to extract mentions of chemicals in the text.

g. Results and Impact The system can transcribe the text, generate meaningful questions, and successfully extract mentions of chemicals.

h. Challenges and Future Work It is difficult to systematically evaluate the performance of this system due to the lack of suitable benchmarks/eval. An obvious extension of this approach is to condition it on further material (e.g., lecture notes and books). In addition, one might automatically score the answers and show them at the beginning and at the end of the video. This would allow us to evaluate the learning of the students and to guide them to the relevant material in case a question was not answered correctly.

V. Meta analysis of the workshop contributions

We have a female/male ratio of about 30 % among the workshop participants who co-authored this paper. We have participants from 22 different institutions in 8 countries.

Most teams combine expertise from different institutions (Figure 21), in several cases beyond academia (Figure 22). Around 20 % of the teams are international, with participants from two countries (Figure 23).

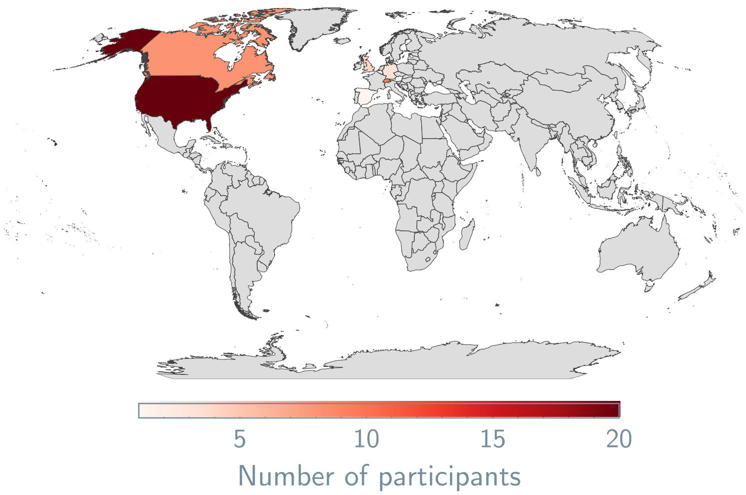

Figure 20. Worldmap (Robin projection) with the number of participants shown in color.

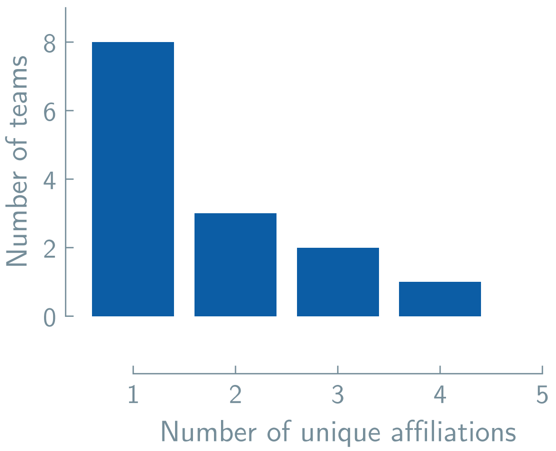

Figure 21. Histogram of the number of unique affiliations per team.

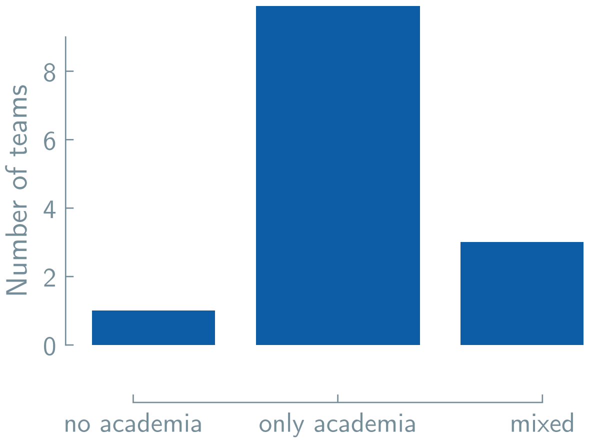

Figure 22. Number of teams with participants only from academia or academia and industry/nonprofit, respectively. We counted national labs as “academia”.

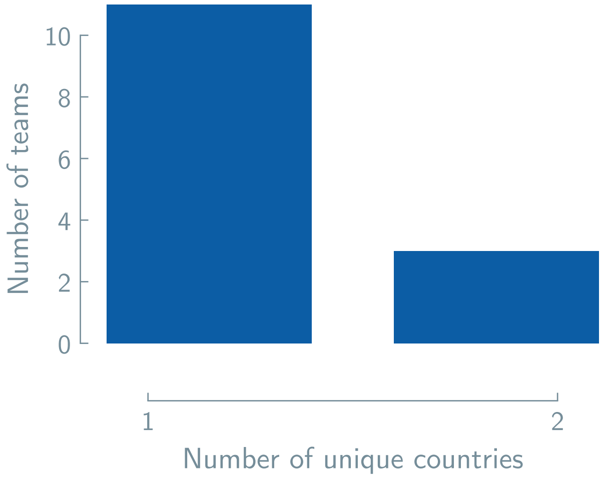

Figure 23. Histogram of the number of unique countries per team.

[1] Ward, L.; Blaiszik, B.; Foster, I.; Assary, R. S.; Narayanan, B.; Curtiss, L. Machine learning prediction of accurate atomization energies of organic molecules from low-fidelity quantum chemical calculations. *MRS Commun.* **2019**, *9*, 891–899.

[2] Curtiss, L. A.; Redfern, P. C.; Raghavachari, K. Gaussian-4 theory using reduced order perturbation theory. *J. Chem. Phys.* **2007**, *127*, 124105.

[3] Ramakrishnan, R.; Dral, P. O.; Rupp, M.; Von Lilienfeld, O. A. Quantum chemistry structures and properties of 134 kilo molecules. *Sci. Data* **2014**, *1*, 1–7.

[4] Narayanan, B.; Redfern, P. C.; Assary, R. S.; Curtiss, L. A. Accurate quantum chemical energies for 133000 organic molecules. *Chem. Sci.* **2019**, *10*, 7449–7455.

[5] Weininger, D. SMILES, a chemical language and information system. 1. Introduction to methodology and encoding rules. *J. Chem. Inf. Comput. Sci.* **1988**, *28*, 31–36.

[6] Krenn, M.; Häse, F.; Nigam, A.; Friederich, P.; Aspuru-Guzik, A. Self-referencing embedded strings (SELFIES): A 100% robust molecular string representation. *Mach. Learn.: Sci. Technol.* **2020**, *1*, 045024.

[7] Krenn, M.; Ai, Q.; Barthel, S.; Carson, N.; Frei, A.; Frey, N. C.; Friederich, P.; Gaudin, T.; Gayle, A. A.; Jablonka, K. M., et al. SELFIES and the future of molecular string representations. *Patterns* **2022**, *3*, 100588.

[8] Jablonka, K. M.; Schwaller, P.; Ortega-Guerrero, A.; Smit, B. Is GPT-3 all you need for low-data discovery in chemistry? *ChemRxiv preprint 10.26434/chemrxiv-2023-fw8n4* **2023**,

[9] Brown, T.; Mann, B.; Ryder, N.; Subbiah, M.; Kaplan, J. D.; Dhariwal, P.; Neelakantan, A.; Shyam, P.; Sastry, G.; Askell, A., et al. Language models are few-shot learners. *Advances in neural information processing systems* **2020**, *33*, 1877–1901.

[10] Ramakrishnan, R.; Dral, P. O.; Rupp, M.; Von Lilienfeld, O. A. Big data meets quantum chemistry approximations: the Δ-machine learning approach. *J. Chem. Theory Comput.* **2015**, *11*, 2087–2096.

[11] Gupta, A. K.; Raghavachari, K. Three-Dimensional Convolutional Neural Networks Utilizing Molecular Topological Features for Accurate Atomization Energy Predictions. *J. Chem. Theory Comput.* **2022**, *18*, 2132–2143.

[12] Mangrulkar, S.; Gugger, S.; Debut, L.; Belkada, Y.; Paul, S. PEFT: State-of-the-art Parameter-Efficient Fine-Tuning methods. `https://github.com/huggingface/peft`, 2022.

[13] Hu, E. J.; Shen, Y.; Wallis, P.; Allen-Zhu, Z.; Li, Y.; Wang, S.; Wang, L.; Chen, W. LoRA: Low-Rank Adaptation of Large Language Models. *arXiv preprint: Arxiv-2106.09685* **2021**,

[14] Radford, A.; Wu, J.; Child, R.; Luan, D.; Amodei, D.; Sutskever, I. Language Models are Unsupervised Multitask Learners. **2019**, `https://d4mucfpksywv.cloudfront.net/better-language-models/language_models_are_unsupervised_multitask_learners.pdf`.

[15] Kojima, T.; Gu, S. S.; Reid, M.; Matsuo, Y.; Iwasawa, Y. Large Language Models are Zero-Shot Reasoners. 2023.

[16] Touvron, H.; Lavril, T.; Izacard, G.; Martinet, X.; Lachaux, M.-A.; Lacroix, T.; Rozière, B.; Goyal, N.; Hambro, E.; Azhar, F., et al. Llama: Open and efficient foundation language models. *arXiv preprint:2302.13971* **2023**,

[17] Lin, Z.; Akin, H.; Rao, R.; Hie, B.; Zhu, Z.; Lu, W.; Smetanin, N.; Verkuil, R.; Kabeli, O.; Shmueli, Y., et al. Evolutionary-scale prediction of atomic-level protein structure with a language model. *Science* **2023**, *379*, 1123–1130.

[18] Andrew, R. Global Co2 Emissions From Cement Production. 2017; `https://zenodo.org/record/831455`.

[19] Lookman, T.; Balachandran, P. V.; Xue, D.; Yuan, R. Active learning in materials science with emphasis on adaptive sampling using uncertainties for targeted design. *npj Comput. Mater.* **2019**, *5*.

[20] Völker, C.; Firdous, R.; Stephan, D.; Kruschwitz, S. Sequential learning to accelerate discovery of alkali-activated binders. *Journal of Materials Science* **2021**, *56*, 15859–15881.

[21] Völker, C.; Benjami Moreno Torres,; Tehseen Rug,; Firdous, R.; Ghezal Ahmad,; Zia, J.; Lüders, S.; Scaffino, H. L.; Höpler, M.; Böhmer, F.; Pfaff, M.; Stephan, D.; Kruschwitz, S. Green building materials: a new frontier in data-driven sustainable concrete design. Preprint 10.13140/RG.2.2.29079.85925. 2023.

[22] Ramos, M. C.; Michtavy, S. S.; Porosoff, M. D.; White, A. D. Bayesian Optimization of Catalysts With In-context Learning. *arXiv preprint: Arxiv-2304.05341* **2023**,

[23] Rao, G. M.; Rao, T. D. G. A quantitative method of approach in designing the mix proportions of fly ash and GGBS-based geopolymer concrete. *Aust. J. Civ. Eng.* **2018**, *16*, 53–63.

[24] OpenAI, Text-davinci-003. `https://platform.openai.com/models/text-davinci-003`.

[25] Bousquet, A. lolopy. `https://pypi.org/project/lolopy/`, 2017; Accessed: 2023-02-27.

[26] Heinisch, O. Steel, R. G. D., and J. H. Torrie: Principles and Procedures of Statistics. (With special Reference to the Biological Sciences.) McGraw-Hill Book Company, New York, Toronto, London 1960, 481 S., 15 Abb. 81 s 6 d. *Biometrische Zeitschrift* **1962**, *4*, 207–208.

[27] Dinh, T.; Zeng, Y.; Zhang, R.; Lin, Z.; Gira, M.; Rajput, S.; Sohn, J.-Y.; Papailiopoulos, D.; Lee, K. LIFT: Language-Interfaced Fine-Tuning for Non-Language Machine Learning Tasks. arXiv preprint: Arxiv-2206.06565. 2022.

[28] Herhold, P.; Farnworth, E. The Net-Zero Challenge: Fast-Forward to Decisive Climate Action. World Economic Forum, available at: https://www3. weforum. org/docs/WEF_The_Net_Zero_ Challenge. pdf (accessed 4 October 2021). 2020.

[29] Hong, Z.; Ajith, A.; Pauloski, G.; Duede, E.; Malamud, C.; Magoulas, R.; Chard, K.; Foster, I. ScholarBERT: Bigger is Not Always Better. arXiv preprint: Arxiv-2205.11342. 2022.

[30] Kim, S.; Thiessen, P. A.; Bolton, E. E.; Chen, J.; Fu, G.; Gindulyte, A.; Han, L.; He, J.; He, S.; Shoemaker, B. A., et al. PubChem substance and compound databases. *Nucleic acids research* **2016**, *44*, D1202–D1213.

[31] Dai, H. et al. AugGPT: Leveraging ChatGPT for Text Data Augmentation. arXiv preprint: Arxiv-2302.13007. 2023.

[32] Wolf, T. et al. Transformers: State-of-the-Art Natural Language Processing. Proceedings of the 2020 Conference on Empirical Methods in Natural Language Processing: System Demonstrations. Online, 2020; pp 38–45.

[33] Pedregosa, F. et al. Scikit-learn: Machine Learning in Python. *Journal of Machine Learning Research* **2011**, *12*, 2825–2830.

[34] Rajpurkar, P.; Jia, R.; Liang, P. Know What You Don't Know: Unanswerable Questions for SQuAD. **2018**,

[35] Zhang, J.; Chang, W.-C.; Yu, H.-F.; Dhillon, I. Fast multi-resolution transformer fine-tuning for extreme multi-label text classification. *Adv. Neural Inf. Process. Syst.* **2021**, *34*, 7267–7280.

[36] White, A. D.; Hocky, G. M.; Gandhi, H. A.; Ansari, M.; Cox, S.; Wellawatte, G. P.; Sasmal, S.; Yang, Z.; Liu, K.; Singh, Y., et al. Assessment of chemistry knowledge in large language models that generate code. *Digital Discovery* **2023**,

[37] Schwaller, P.; Laino, T.; Gaudin, T.; Bolgar, P.; Hunter, C. A.; Bekas, C.; Lee, A. A. Molecular transformer: A model for uncertainty-calibrated chemical reaction prediction. *ACS Central Science* **2019**, *5*, 1572–1583.

[38] Schwabe, T.; Grimme, S. Theoretical thermodynamics for large molecules: walking the thin line between accuracy and computational cost. *Acc. Chem. Res.* **2008**, *41*, 569–579.

[39] Skyner, R. E.; McDonagh, J. L.; Groom, C. R.; van Mourik, T.; Mitchell, J. B. O. A review of methods for the calculation of solution free energies and the modelling of systems in solution. *Phys. Chem. Chem. Phys.* **2015**, *17*, 6174–6191.

[40] Schleder, G. R.; Padilha, A. C. M.; Acosta, C. M.; Costa, M.; Fazzio, A. From DFT to machine learning: recent approaches to materials science–a review. *J. Phys. Mater.* **2019**, *2*, 032001.

[41] Chase, H. LangChain. 2022; `https://github.com/hwchase17/langchain`.

[42] Bran, A. M.; Cox, S.; White, A. D.; Schwaller, P. ChemCrow: Augmenting large-language models with chemistry tools. *arXiv preprint: Arxiv-2304.05376* **2023**,

[43] Jain, A.; Ong, S. P.; Hautier, G.; Chen, W.; Richards, W. D.; Dacek, S.; Cholia, S.; Gunter, D.; Skinner, D.; Ceder, G.; Persson, K. A. Commentary: The Materials Project: A materials genome approach to accelerating materials innovation. *APL Materials* **2013**, *1*, 011002.

[44] McDermott, M. J.; Dwaraknath, S. S.; Persson, K. A. A Graph-Based Network for Predicting Chemical Reaction Pathways in Solid-State Materials Synthesis. *Nat. Commun.* **2021**, *12*, 3097.

[45] Shao, Z.; Gong, Y.; Shen, Y.; Huang, M.; Duan, N.; Chen, W. Synthetic Prompting: Generating Chain-of-Thought Demonstrations for Large Language Models. **2023**,

[46] Gao, L.; Schulman, J.; Hilton, J. Scaling Laws for Reward Model Overoptimization. *ARXIV.ORG* **2022**,

[47] Rego, N.; Koes, D. 3Dmol.js: molecular visualization with WebGL. *Bioinformatics* **2014**, *31*, 1322–1324.

[48] Schrödinger, L.; DeLano, W. PyMOL. `http://www.pymol.org/pymol`.

[49] Sehnal, D.; Bittrich, S.; Deshpande, M.; Svobodová, R.; Berka, K.; Bazgier, V.; Velankar, S.; Burley, S. K.; Koča, J.; Rose, A. S. Mol* Viewer: modern web app for 3D visualization and analysis of large biomolecular structures. *Nucleic Acids Res.* **2021**, *49*, W431–W437.

[50] Yao, S.; Zhao, J.; Yu, D.; Du, N.; Shafran, I.; Narasimhan, K.; Cao, Y. ReAct: Synergizing Reasoning and Acting in Language Models. *arXiv preprint: Arxiv-2210.03629* **2023**,

[51] Thompson, A. P.; Aktulga, H. M.; Berger, R.; Bolintineanu, D. S.; Brown, W. M.; Crozier, P. S.; in 't Veld, P. J.; Kohlmeyer, A.; Moore, S. G.; Nguyen, T. D.; Shan, R.; Stevens, M. J.; Tranchida, J.; Trott, C.; Plimpton, S. J. LAMMPS - a flexible simulation tool for particle-based materials modeling at the atomic, meso, and continuum scales. *Comp. Phys. Comm.* **2022**, *271*, 108171.

[52] Abraham, M. J.; Murtola, T.; Schulz, R.; Páll, S.; Smith, J. C.; Hess, B.; Lindahl, E. GROMACS: High performance molecular simulations through multi-level parallelism from laptops to supercomputers. *SoftwareX* **2015**, *1-2*, 19–25.

[53] Volk, A. A.; Epps, R. W.; Yonemoto, D. T.; Masters, B. S.; Castellano, F. N.; Reyes, K. G.; Abolhasani, M. AlphaFlow: autonomous discovery and optimization of multi-step chemistry using a self-driven fluidic lab guided by reinforcement learning. *Nat. Commun.* **2023**, *14*, 1403.

[54] Griffiths, R.-R. et al. GAUCHE: A Library for Gaussian Processes in Chemistry. 2022; `http://arxiv.org/abs/2212.04450`, arXiv:2212.04450 [cond-mat, physics:physics].

[55] Shields, B. J.; Stevens, J.; Li, J.; Parasram, M.; Damani, F.; Alvarado, J. I. M.; Janey, J. M.; Adams, R. P.; Doyle, A. G. Bayesian reaction optimization as a tool for chemical synthesis. *Nature* **2021**, *590*, 89–96.

[56] Ranković, B.; Griffiths, R.-R.; Moss, H. B.; Schwaller, P. Bayesian optimisation for additive screening and yield improvements in chemical reactions – beyond one-hot encodings. ChemRxiv preprint 10.26434/chemrxiv-2022-nll2j. 2022.

[57] Neo4j, Neo4j - The World's Leading Graph Database. 2012; http://neo4j.org/.

[58] Venugopal, V.; Pai, S.; Olivetti, E. MatKG: The Largest Knowledge Graph in Materials Science–Entities, Relations, and Link Prediction through Graph Representation Learning. *arXiv preprint:2210.17340* **2022**,

[59] McCusker, J. P.; Deagen, M.; Fateye, T.; Wallace, A.; Rashid, S. M.; McGuinness, D. L. Creating and Visualizing the Materials Science Knowledge Graph with Whyis. ISWC (Posters/Demos/Industry). 2021.

[60] Dunn, A.; Dagdelen, J.; Walker, N.; Lee, S.; Rosen, A. S.; Ceder, G.; Persson, K. A.; Jain, A. Structured information extraction from complex scientific text with fine-tuned large language models. *arXiv preprint: Arxiv-2212.05238* **2022**,

[61] Badhwar, S. Smart Manufacturing - A Case for Creating a Knowledge Network Using Data Mining. 2022.

[62] McCusker, J. P.; Keshan, N.; Rashid, S.; Deagen, M.; Brinson, C.; McGuinness, D. L. NanoMine: A knowledge graph for nanocomposite materials science. The Semantic Web–ISWC 2020: 19th International Semantic Web Conference, Athens, Greece, November 2–6, 2020, Proceedings, Part II. 2020; pp 144–159.

[63] Kearnes, S. M.; Maser, M. R.; Wleklinski, M.; Kast, A.; Doyle, A. G.; Dreher, S. D.; Hawkins, J. M.; Jensen, K. F.; Coley, C. W. The Open Reaction Database. *J. Am. Chem. Soc. 143*, 18820–18826.

[64] Taori, R.; Gulrajani, I.; Zhang, T.; Dubois, Y.; Li, X.; Guestrin, C.; Liang, P.; Hashimoto, T. B. Stanford Alpaca: An Instruction-following LLaMA model. https://github.com/tatsu-lab/stanford_alpaca, 2023.

[65] Alpaca-LoRA. https://github.com/tloen/alpaca-lora.

[66] Colter, Z.; Fayazi, M.; Youbi, Z. B.-E.; Kamp, S.; Yu, S.; Dreslinski, R. Tablext: A combined neural network and heuristic based table extractor. *Array* **2022**, *15*, 100220.

[67] Mamaghani, Z. G.; Hawboldt, K. A.; MacQuarrie, S. Adsorption of CO_2 using biochar - Review of the impact of gas mixtures and water on adsorption. *J. Environ. Chem. Eng.* **2023**, *11*, 109643.

[68] Peng, Y.; Krungleviciute, V.; Eryazici, I.; Hupp, J. T.; Farha, O. K.; Yildirim, T. Methane Storage in Metal–Organic Frameworks: Current Records, Surprise Findings, and Challenges. *J. Am. Chem. Soc.* **2013**, *135*, 11887–11894.

[69] Sahoo, B.; Pandey, V.; Dogonchi, A.; Mohapatra, P.; Thatoi, D.; Nayak, N.; Nayak, M. A state-of-art review on 2D material-boosted metal oxide nanoparticle electrodes: Supercapacitor applications. *J. Energy Storage* **2023**, *65*, 107335.

[70] Suppiah, D. D.; Daud, W. M. A. W.; Johan, M. R. Supported Metal Oxide Catalysts for CO_2 Fischer–Tropsch Conversion to Liquid Fuels-A Review. *Energy Fuels.* **2021**, *35*, 17261–17278.

[71] González-Vázquez, M.; García, R.; Gil, M.; Pevida, C.; Rubiera, F. Comparison of the gasification performance of multiple biomass types in a bubbling fluidized bed. *Energy Convers. Manag.* **2018**, *176*, 309–323.

[72] Mohsin, M.; Farhan, S.; Ahmad, N.; Raza, A. H.; Kayani, Z. N.; Jafri, S. H. M.; Raza, R. The electrochemical study of $NixCe_{1-x}O_{2-\delta}$ electrodes using natural gas as a fuel. *New J. Chem.* **2023**, *47*, 8679–8692.

[73] Kaur, P.; Singh, K. Review of perovskite-structure related cathode materials for solid oxide fuel cells. *Ceram. Int.* **2020**, *46*, 5521–5535.

[74] Sengottuvelu, R. jsonformer. https://github.com/1rgs/jsonformer, 2018.

[75] Choudhary, K.; Kelley, M. L. ChemNLP: A Natural Language Processing based Library for Materials Chemistry Text Data. *arXiv preprint arXiv:2209.08203* **2022**,

[76] Kunc, K.; Loa, I.; Syassen, K.; Kremer, R.; Ahn, K. MgB2 under pressure: phonon calculations, Raman spectroscopy, and optical reflectance. *arXiv preprint cond-mat/0105402*

[77] FameLab International — Cheltenham Festivals. https://www.cheltenhamfestivals.com/famelab,last accessed 2023-05-30.

[78] MT 180 - My Thesis in 180 Seconds. https://www.epfl.ch/campus/events/events/public-events/my-thesis-in-180-seconds,last accessed 2023-07-07.

[79] ClIPDIGEST. https://clipdigest.com/, last accessed 2023-05-30.

[80] Radford, A.; Kim, J. W.; Xu, T.; Brockman, G.; McLeavey, C.; Sutskever, I. Robust speech recognition via large-scale weak supervision. arXiv preprint: ArXiv-2212.04356. 2022.

[81] Kim, S.; Chen, J.; Cheng, T.; Gindulyte, A.; He, J.; He, S.; Li, Q.; Shoemaker, B. A.; Thiessen, P. A.; Yu, B.; Zaslavsky, L.; Zhang, J.; Bolton, E. E. PubChem 2023 update. *Nucleic Acids Res.* **2022**, *51*, D1373–D1380.

[82] Kim, S.; Chen, J.; Cheng, T.; Gindulyte, A.; He, J.; He, S.; Li, Q.; Shoemaker, B. A.; Thiessen, P. A.; Yu, B.; Zaslavsky, L.; Zhang, J.; Bolton, E. E. PubChem 2019 update: improved access to chemical data. *Nucleic Acids Res.* **2018**, *47*, D1102–D1109.

[83] Kim, S.; Thiessen, P. A.; Cheng, T.; Yu, B.; Bolton, E. E. An update on PUG-REST: RESTful interface for programmatic access to PubChem. *Nucleic Acids Res.* **2018**, *46*, W563–W570.

[84] Streamlit. https://streamlit.io/.

Acronyms

AI: artificial intelligence.

API: application programming interface.

BO: Bayesian optimization.

CAS: Chemical Abstract Services.

COT: chain of thought.

DFT: density functional theory.

DOI: digital object identifier.

ELN: electronic lab notebook.

GA: genetic algorithm.

GPR: Gaussian process regression.

GPT: generative pretrained transformer.

GUI: graphical user interface.

HTML: HyperText Markup Language.

ICL: in-context learning.

ID: inverse design.

InChI: international chemical identifier.

JSON: JavaScript object notation.

LIFT: language-interfaced fine-tuning.

LIMS: laboratory information system.

LLM: large language model.

LoRA: low-rank adaptors.

MAD: median absolute deviation.

MAE: mean absolute error.

MAPI: Materials Project API.

ML: machine learning.

NER: named entity recognition.

NLM: national library of medicine.

NLP: natural language processing.

OCR: optical character recognition.

ORD: Open Reaction Database.

PDB: protein data bank.

PEFT: parameter efficient fine-tuning.

RF: random forest.

RLHF: reinforcement learning from human feedback.

ROUGE: Recall-Oriented Understudy for Gisting Evaluation.

SELFIES: self-referencing embedded strings.

SMILES: simplified molecular-input line-entry system.

SVM: support vector machine.

UI: user interface.
